# Closer vein spacing by ectopic expression of nucleotide-binding and leucine-rich repeat proteins in rice leaves

**DOI:** 10.1007/s00299-021-02810-5

**Published:** 2021-11-27

**Authors:** Shuen-Fang Lo, Jolly Chatterjee, Akshaya K. Biswal, I.-Lun Liu, Yu-Pei Chang, Pei-Jing Chen, Samart Wanchana, Abigail Elmido-Mabilangan, Robert A. Nepomuceno, Anindya Bandyopadhyay, Yue-Ie Hsing, William Paul Quick

**Affiliations:** 1 Biotechnology Center, National Chung Hsing University, Taichung, 402 Taiwan, ROC; 2 C4 Rice Centre, International Rice Research Institute (IRRI), Los Baños, Philippines; 3 Genetic Resources Program, International Maize and Wheat Improvement Center (CIMMYT), Carretera México-Veracruz km. 45, El Batán, Texcoco, CP 56237 México; 4 National Institute of Molecular Biology and Biotechnology, University of the Philippines (BIOTECH-UPLB), Los Baños, 4031 Philippines; 5 R&D Synthetic Biology, Reliance Industries Ltd, Mumbai, India; 6 Institute of Plant and Microbial Biology, Academia Sinica, Taipei, 115 Taiwan, ROC; 7 Department of Animal and Plant Sciences, University of Sheffield, Sheffield, UK

**Keywords:** Close vein spacing (CVS), Vein density (VD), Nucleotide-binding and leucine-rich repeat (NB-LRR) protein, Rice, Taiwan Rice Insertional Mutants (TRIM) population

## Abstract

**Key message:**

Elevated expression of nucleotide-binding and leucine-rich repeat proteins led to closer vein spacing and higher vein density in rice leaves.

**Abstract:**

To feed the growing global population and mitigate the negative effects of climate change, there is a need to improve the photosynthetic capacity and efficiency of major crops such as rice to enhance grain yield potential. Alterations in internal leaf morphology and cellular architecture are needed to underpin some of these improvements. One of the targets is to generate a “Kranz-like” anatomy in leaves that includes decreased interveinal spacing close to that in C_4_ plant species. As C_4_ photosynthesis has evolved from C_3_ photosynthesis independently in multiple lineages, the genes required to facilitate C_4_ may already be present in the rice genome. The Taiwan Rice Insertional Mutants (TRIM) population offers the advantage of gain-of-function phenotype trapping, which accelerates the identification of rice gene function. In the present study, we screened the TRIM population to determine the extent to which genetic plasticity can alter vein density (VD) in rice. Close vein spacing mutant 1 (*CVS1*), identified from a VD screening of approximately 17,000 TRIM lines, conferred heritable high leaf VD. Increased vein number in *CVS1* was confirmed to be associated with activated expression of two nucleotide-binding and leucine-rich repeat (NB-LRR) proteins. Overexpression of the two *NB-LRR* genes individually in rice recapitulates the high VD phenotype, due mainly to reduced interveinal mesophyll cell (M cell) number, length, bulliform cell size and thus interveinal distance. Our studies demonstrate that the trait of high VD in rice can be achieved by elevated expression of NB-LRR proteins limited to no yield penalty.

**Supplementary Information:**

The online version contains supplementary material available at 10.1007/s00299-021-02810-5.

## Introduction

Rice is a major staple crop that feeds more of the human population than any other crop. The rice yield needs to be significantly increased to secure food supplies in the next few decades. As a C_3_ plant, rice productivity has reached a ceiling due to its inferior photosynthetic capacity to harvest sunlight, and traditional breeding methods have difficulty achieving a substantial increase in food production. One important strategy to significantly enhance rice productivity is to introduce C_4_ photosynthesis into rice, as C_4_ crops have higher photosynthetic capacity, reduced water loss, increased nitrogen (N) use efficiency and higher yields, particularly when grown in hot and dry environments (Furbank et al. 2009; Hibberd et al. 2008). Recent developments in engineering C_4_ photosynthesis into rice to promote photosynthetic efficiency and yield potential have led to renewed interest in this area (Ermakova et al. 2020; Hibberd et al. 2008; von Caemmerer et al. 2012a, b). However, introducing the C_4_ trait into rice is a highly challenging project and requires multifaceted modifications to leaf development and metabolism (Kumar and Kellogg 2019; Sedelnikova et al. 2018). The alteration of internal leaf architecture is one of the key prerequisites for establishing the “Kranz anatomy” (Lundgren et al. 2014; Sage et al. 2014).

Close vein spacing with Kranz anatomy in leaves is a distinctive feature of the majority of C_4_ plant species (Kajala et al. 2011; Kumar and Kellogg 2019). Kranz anatomy is generally composed of a double concentric layer of chlorenchyma cells. The outer mesophyll (M) cells are positioned close to the intercellular air spaces, and the inner specialized bundle sheath (BS) cells surrounding veins are positioned adjacent to M cells in leaves. Such an anatomical arrangement allows the CO_2_ fixation and decarboxylation steps of photosynthesis to be compartmentalized within the two distinct cell types, M and BS cells, respectively. An increased frequency of veins per unit leaf area in C_4_ plants reduces the space between two veins and facilitates the rapid transport of metabolites between M and BS cells (Langdale and Nelson 1991). While the physiology and biochemistry of C_4_ photosynthesis are well known, the genetic basis of Kranz anatomy remains largely unknown.

In addition to being an imperative part of C_4_ anatomy, vascular tissues function as circulatory organs for supplying water and nutrients to the plant. Vascular tissues are present throughout the plant body from the shoot tip to the root tip (Scarpella and Meijer 2004). Increased VD also has physiological benefits including better hydraulic performance to keep leaves cool and to support photosynthesis in a warm climate. In dicot leaves, veins run in every direction, forming a complicated network, whereas veins are arranged in parallel in monocot leaves and follow basipetal (from tip to base) and acropetal (from base to tip) developmental patterns (Sedelnikova et al. 2018). In monocots, there are three types of longitudinal veins of leaves: the midrib, large veins and small veins (Sack and Scoffoni 2013). Large and small veins are connected by several lateral commissural veins. Molecular and genetic studies on C_4_ plants, mostly in maize and sorghum, have provided insights into the function, regulation and biological consequences of vein pattern modification in monocots (Kumar and Kellogg 2019). These studies reveal that vein development is regulated by a complex interplay among the hormones auxin and brassinosteroid and the transcription factors *SHORTROOT 1 (SHR1)/SCARECROW 1 (SCR1)* and *INDETERMINATE DOMAIN* (IDD) (Kumar and Kellogg 2019; Linh et al. 2018; Sedelnikova et al. 2018). Several mutants with defects or improvements in vein and BS cell development, have been identified in rice (Feldman et al. 2014, 2017; Scarpella et al. 2003; Smillie et al. 2012), which suggests that rice does possess genetic plasticity for altering vein spacing.

A major requirement for engineering Kranz anatomy in a C_3_ leaf would be a decrease in the BS-to-M cell ratio, ideally accomplished by increasing the number of veins to effectively increase the BS cell area and decrease the M cell area (Langdale 2011; Sage et al. 2014; Sedelnikova et al. 2018). Since C_4_ photosynthesis occurred via a series of evolutionary modifications from C_3_ photosynthesis on multiple independent occasions over the last 30 million years (Sage et al. 2011), it is likely that rice already contains all the genes required to induce these changes. Introduction of C_4_ genes into C_3_ plants, pyramiding C_4_-specific genes in one plant, and loss or editing of genes of ancestral C_3_ genes are feasible approaches to C_4_ evolution (Clayton et al. 2017; Peng and Zhang 2021; Schuler et al. 2016; Sen et al. 2017; Wang et al. 2016a, b). The rice leaf anatomy is intermediate between these anatomy of most C_3_ and C_4_ grasses, indicating that the introduction of Kranz anatomy into rice may not require radical changes (Sage and Sage 2009). However, it is unclear whether the rice genome possesses sufficient “plasticity” with respect to the alteration in leaf morphology that is required to raise photosynthetic rates.

As a first step toward manipulation of rice leaf architecture to phenocopy, a Kranz anatomy into rice, this study set out to identify relevant mutants and genes governing the change in VD in rice by screening a large mutant population. Insertional mutagenesis, whereby T-DNA vectors containing multimeric *CaMV35S* enhancers are inserted randomly into the rice genome, can be used to activate gene expression, thereby leading to gain-of-function mutations (Hsing et al. 2007; Lo et al. 2016). Screening these mutant populations will facilitate the identification of the missing genetic components regulating the C_4_ Kranz anatomy.

Members of the nucleotide-binding and leucine-rich repeat (NB-LRR) protein family have been found to serve as crucial regulators of inflammatory and innate immune responses in animals and plants, respectively (Ye and Ting 2008). The majority of plant disease resistance (R) proteins conferring resistance to bacterial, fungal, oomycete or viral pathogens encode proteins belonging to the NB-LRR protein family (Dangl and Jones 2001). The central NB domain has a role in signal transduction mediated by nucleotide phosphorylation and is the most conserved part of the gene; the C-terminal LRR domain is generally required for specific recognition of pathogen effectors; and the N-terminal coiled-coil (CC) domain is present only in NB-LRRs from monocots involved in signaling and likely pathogen recognition (Takken and Goverse 2012). The Arabidopsis and rice genomes contain 150 and 480 *NB-LRR* genes, respectively (Yang et al. 2006) and to date, most of them have not been studied. Ectopic expression of several *CC-NB-LRR* and *NB-LRR* genes isolated from Arabidopsis, maize and rice can confer resistance to blast disease caused by *Magnaporthe oryzae* in rice (Li et al. 2019; Ma et al. 2015; Singh et al. 2020; Xu et al. 2018), indicating the functional conservation of NB-LRRs against pathogens.

We screened a total of approximately 17,000 TRIM lines for alterations in vein patterning and leaf cellular architecture. Using a simple, high-throughput screen for leaf VD, we identified mutant lines with a heritable increase in the number of veins per unit leaf width due to a reduction in the interveinal distance, a phenotype designated as close vein spacing (CVS). Part of the TRIM population was screened previously along with rice variety IR64 deletion mutants to determine the range of VD in rice, although the causal genes were never identified (Feldman et al. 2014). In the present study, *CVS* mutants from the TRIM population were identified and characterized in terms of their potential to engineer C_4_ leaf anatomy in rice. We further demonstrated that ectopic expression of each of two *NB-LRR* and *CC-NB-LRR* genes makes the interveinal distance closer by reducing the interveinal M cell number, M cell length and bulliform cell size, leading to increased VD in rice leaves. The increase in VD in *G2-NB-LRR* and *G7-NB-LRR* transgenic plants was highest at the seedling stage and became insignificant when the plants entered the reproductive stage, which avoided adverse effects on grain yield. To the best of our knowledge, this is the first report on the function of *NB-LRR* genes in the regulation of leaf internal architecture.

## Materials and methods

### Plant materials

For gene expression analysis and seedling morphology characterization, seeds were surface sterilized in 2.5% sodium hypochlorite and germinated on half-strength MS agar medium (Murashige and Skoog Basal Medium with Vitamins; Phyto Technology Laboratories®) (Murashige and Skoog 1962) at 28 °C with 16 h of light and 8 h of darkness for 10–20 days. For yield analysis and vein spacing evaluation, plants were cultivated at the National Chung-Hsing University experimental farm under natural growing conditions.

### Screening for leaf *CVS* mutants

A total of 17,324 T_1_ generation lines with 12 plants per line were screened from the Taiwan Rice Insertional Mutants (TRIM) population (http://rice.sinica.edu.tw/fgb2/gbrowse/TRIM_gb/) in 8 field experiments at either National Chung Hsing University, Taiwan (25.0330° N, 121.5654° E) or the International Rice Research Institute (IRRI) in the Philippines (14° 9′ 53.58″ N 121° 15′ 32.19″ E) from 2009 to 2012 (Supplementary Table S1). The population used T-DNA *pTAG8* containing an enhancer –tetramer and selectable markers (Supplementary Fig. S1), which function in gene trapping, knockout and activation tagging (Hsing et al. 2007; Lo et al. 2016) in the genetic background of *Oryza sativa* cv Tainung 67 (TNG67).

Sterilized seeds were germinated on sterile damp filter paper in Petri dishes in the dark at 30 °C for 3 days, followed by 2 days in the light at the same temperature. Seedlings were transplanted into pots maintained in a screen house or in the field. Pots were filled with soil from the IRRI upland farm mixed with 25% coco-coir and 0.4 g/L Osmocote Plus 15-9-12 (The Scotts Company Ltd., Thorne, UK). One of the high VD candidate mutants, M0104656, was grown in successive generations (*T*_2_–*T*_7_) in pots with soil in the screen house at IRRI.

Leaf VD is defined as the total number of veins per mm leaf width. A 5-cm-long piece of the mid-section of the fully expanded fifth leaf was sampled for VD quantification. VD was counted in a 1-mm field of view at four locations on both the left- and right-hand sides of the leaf using a Meade ReadView Portable Microscope (Meade Instruments Corp. CA, USA). For experiments conducted in Taiwan, leaf samples were fixed in formaldehyde alcohol fixatives and imported to IRRI. The VD of each line was counted in ImageJ and recorded in a Microsoft Excel Workbook (Microsoft Corp, USA). Any mutant plant found to have > 7.0 veins in a 1-mm field of view was considered to have a CVS phenotype and was subjected to detailed microscopic examination. Mutant lines with a heritable CVS phenotype were further characterized in successive generations.

### Leaf anatomy

Leaves were examined using cleared sections, thin sections or fluorescence images to detect chloroplast positions. Fluorescence images of leaf cross-sections were captured using fresh leaves to detect chloroplast positions as described (Chatterjee et al. 2016). Leaves were fixed in FAA solution [3.7% (v/v) formaldehyde, 5% (v/v) acetic acid, and 50% ethanol] and were later used for preparation of cleared sections. Leaf sections were cleared as described (Lux et al. 2005) and stained with 0.05% toluidine blue.

For a detailed characterization of leaf anatomy, thin sections were prepared from leaves fixed in a 2.5% glutaraldehyde solution as described (Chatterjee et al. 2016). Leaf discs were dehydrated in a graded ethanol series (McKown and Dengler 2007) and embedded in Spurr’s resin (Spurr 1969). Samples were sectioned using a Sorvall MT2-B Ultramicrotome (DuPont-Instruments-Sorvall, Newtown, CT, USA) and stained in 0.05% toluidene blue. All sections were viewed under an OLYMPUS BX51 or motorized BX61 and/or BX63 microscope (Olympus Optical, Tokyo, Japan). Leaf section images were acquired with an Olympus DP71 digital documentation system attached to the microscope.

### Image analysis

All images of leaf anatomy were analyzed with Olympus cellSens software (www.olympus-lifescience.com/en/software/cellsens/) and ImageJ software v.1.43 (https://imagej.nih.gov/ij/index.html) to determine leaf VD, leaf thickness (µm), interveinal distance (µm), M cell length (µm), M cell number between two minor veins, M cell total area (mm^2^), M cell lobing (the ratio of the actual cell perimeter to the minimum circumference of the cell), BS cell number, BS cell area (µm^2^), vein area (µm^2^) and bulliform cell area (µm^2^). Measurements were made only at the middle portion of transverse leaf sections. M cell length and lobing were examined as described (Chatterjee et al. 2016; Giuliani et al. 2013). Measurements were made on 25 random segments from 3 sections per leaf and 3 leaves from 3 plants per line. Leaf width (mm) was measured prior to leaf sectioning.

### Gas exchange measurements

Leaf gas exchange measurements were made at IRRI (mean atmospheric pressure of 94.8 kPa) using a Li-6400XT infrared gas exchange analyzer (LI-COR Biosciences, Lincoln, NE, USA) fitted with a standard 2 × 3 cm leaf chamber and 6400-02 B light source. Measurements were made at a constant airflow rate of 400 μmol s^−1^, leaf temperature of 30 °C, leaf-to-air vapor deficit between 1.0 and 1.5 kPa and relative humidity of 60–65%. Data were acquired between 0800 and 1300 h in a room with the air temperature maintained at approximately 30 °C. Measurements were made on the mid-portion of the leaf blade of three fully expanded leaves formed during the tillering stage from two plants. Leaves were acclimated in the cuvette for approximately 30 min before measurements were made. The response curves of the net rate of assimilation (*A*, µmol m^−2^ s^−1^) to changing intercellular CO_2_ concentration (*Ci*, µmol CO_2_ mol^−1^) were acquired by increasing the *Ca* (CO_2_ concentration in the cuvette) from 20 to 1500 µmol CO_2_ mol air^−1^ at a photosynthetic photon flux density (PPFD) of 1000 µmol photon m^−2^ s^−1^. Light response curves were acquired by decreasing the PPFD from 2000 to 0 µmol photons m^−2^ s^−1^ at *Ca* 400 µmol CO_2_ mol^−1^. The CO_2_ compensation point (*Γ*) and maximum carboxylation efficiency (*CE*) were calculated from the intercept (Vogan et al. 2007) and slope (Wang et al. 2006) of the CO_2_ response curves. The quantum yield for CO_2_ assimilation (φ) was calculated from the slope of the light response curves (Farquhar and Wong 1984). The maximum carboxylation rate allowed by Rubisco (*V*_cmax_), rate of photosynthetic electron transport based on NADPH requirements (*J*), triose phosphate use (*TPU*), daytime respiration (*R*_d_) and mesophyll conductance (*g*_m_) were calculated using the curve fitting tool as described (Sharkey et al. 2007).

### T-DNA flanking sequence analysis

Genomic DNA of mutants was extracted with CTAB extraction buffer as described (Doyle 1987). T-DNA flanking sequences were recovered using a built-in plasmid rescue system (Upadhyaya et al. 2002) and analyzed with an ABI Prism 3100 DNA sequencer (Applied Biosystems) using DNA sequences 100 bp upstream of the T-DNA right border (Hsing et al. 2007) as an RB primer (Supplementary Table S2). T-DNA flanking sequences were blasted against the Rice Annotation Project Database (RAP-DB, https://rapdb.dna.affrc.go.jp/viewer/gbrowse/irgsp1/) or MSU Rice Genome Annotation Project 7 (RGAP 7, http://rice.plantbiology.msu.edu/) (Kawahara et al. 2013) for identification of the T-DNA insertion site. Gene loci within a 40-kb region up- and downstream of the T-DNA insertion site were obtained from the RAP-DB or RGAP 7 database.

For analysis of the T-DNA copy number in *CVS1* and the T-DNA insertion site that associates with the CVS phenotype, genomic DNA was extracted from leaves of *CVS1*, digested with *Sph I*, and subjected to DNA gel blot analysis using the hygromycin phosphotransferase gene (*Hyg*) as a probe.

### Quantitative RT-PCR

Total RNA was extracted from the first fully expanded leaf of the main tiller of two plants per line using TRIzol Reagent (Thermo Fisher Scientific, USA). Real-time polymerase chain reaction (RT-PCR) analyses were conducted as described (Lo et al. 2008).

### Statistical analysis

All statistical analyses were performed in STAR, an R-based software developed by IRRI or with Student’s *t* test using SigmaPlot software (version 11.0, Systat Software, Inc.). All results are presented as the mean ± SE. Significance levels were determined with the *t* test: *P < 0.05, **P < 0.01, ***P < 0.001.

### Transgenic rice overexpressing *NB-LRR*

Full-length cDNAs of genes flanking the T-DNA insertion site in *CVS1* were PCR-amplified from rice (TNG67) mRNA based on their putative open reading frames annotated with the RGAP 7 database (Kawahara et al. 2013). cDNAs were ligated into the pGEM®-T Easy cloning vector (Promega), and their sequences were confirmed by DNA sequencing analysis. Plasmid *pAHC18* (Bruce et al. 1989), derived from plasmid *pUC18*, contains the maize ubiquitin gene (*Ubi*) promoter and nopaline synthase gene (*Nos*) terminator. cDNAs were excised from the pGEM-T Easy vector and ligated into a site between the *Ubi* promoter and *Nos* terminator in plasmid pAHC18. Plasmids containing Ubidriven cDNA of various genes were individually linearized with *HindIII* and inserted into the same site in pCAMBIA1301 (Hajdukiewicz et al. 1994). The resulting binary vectors were transferred into *Agrobacterium tumefaciens* strain EHA105. Calli were induced from immature rice seeds of *Oryza sativa* cv Tainung 67 for rice transformation. The calli were cocultured with *A. tumefaciens* with binary vectors. The *T*_0_ transgenic plants were regenerated and screened from calli following the method described (Chen et al. 2002).

To calculate the vein density of transgenic plants in various generations, we screened out the segregated wild type of transgenic plants. All analyzed plants were heterozygous/homozygous, and the uppermost fully expanded leaf was collected for all VD data calculations.

### Phylogenetic analysis of NB-LRRs

Phylogenetic analysis of NB-LRRs in rice and other plant species was performed with full-length amino acid sequences by MEGA X software (Kumar et al. 2018) using the neighbor-joining method (Saitou and Nei 1987). The evolutionary distance was computed using the Poisson correction method (Zuckerkandl and Pauling 1965) and is reported as the number of amino acid substitutions per site. All ambiguous positions were removed for each sequence pair (pairwise deletion option). The accession numbers of the genes are listed in Supplementary Table S5.

### Primers

All primers used for DNA sequencing, quantitative RT-PCR and genome typing are provided in Supplementary Table S2.

## Results

### Screening and identification of the *CVS1* mutant

**Fig. 1:**
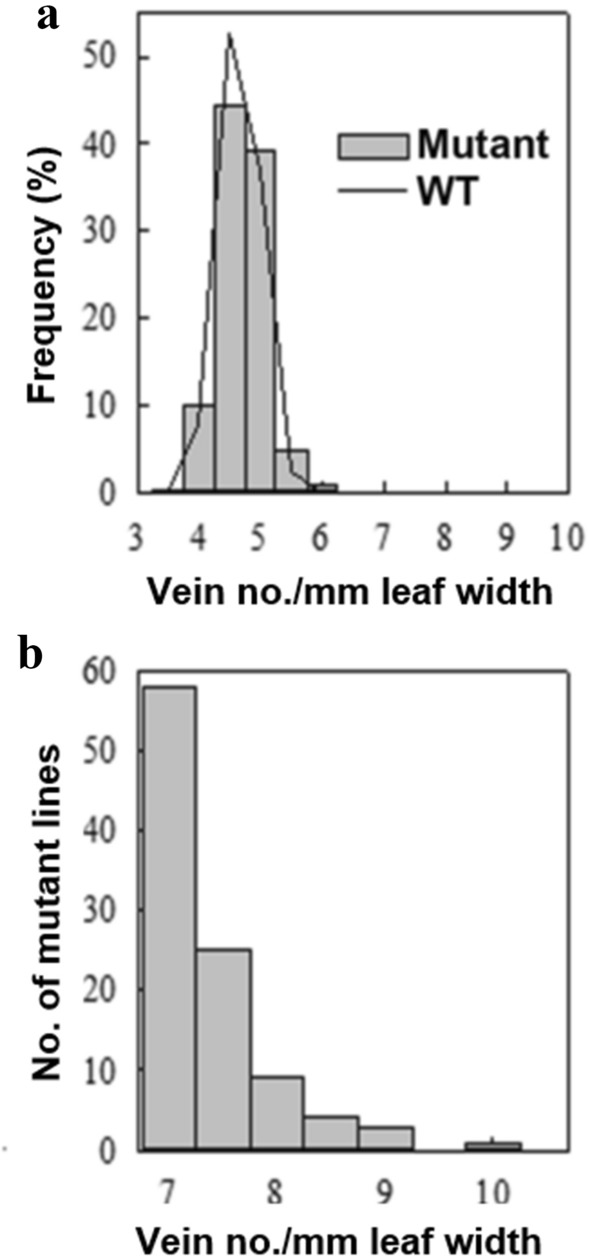
VD screening of the TRIM population for high VD mutants and frequency of VD distribution in mutants. **a** Frequency of VD (vein number per mm leaf width) distribution in WT (*n* = 3139) and mutant lines (*n* = 17,324). **b** Number of mutant lines (*n* = 100) with VD equal to or greater than 7 veins per mm leaf width

The VD of wild-type (WT) plants ranged between 4 and 6.5 veins per mm of leaf width, with 84% of the population having a VD of either 4.5 or 5.0 veins per mm (Fig. 1a). The range of VD was broader in the mutant population, numbering between 2.5 and 10 veins per mm (Fig. 1a). Approximately, 0.32% of the mutant population had a VD lower than 4 veins per mm, and only 0.05% had a VD of 7 or more veins per mm leaf width.

The threshold VD for a mutant to be considered as a CVS candidate was set at 7 veins per mm leaf width, which is above the maximum VD of the WT population. From a total of 17,324 TRIM lines screened (Supplementary Table S1), 100 candidates were identified as having the CVS phenotype (Fig. 1b). A total of 49 candidate lines were prioritized for secondary screening by selecting only those with the CVS phenotype on both sides of the leaf. Of these, 23 mutant lines could not be rescreened, as the CVS phenotype was associated with seed sterility or was lethal. Only 7 of the 26 remaining lines showed a heritable phenotype in the *T*_2_ generation, and only 3 in the *T*_3_ generation exhibited the phenotype (Supplementary Table S3). Due to low yield in one of the three mutant lines (M0110124), only two *CVS* mutants, M0104656 (*CVS1*) and M0105588 (*CVS2*), were screened beyond the *T*_4_ generation. *CVS1* was further characterized in this study.

**Fig. 2:**
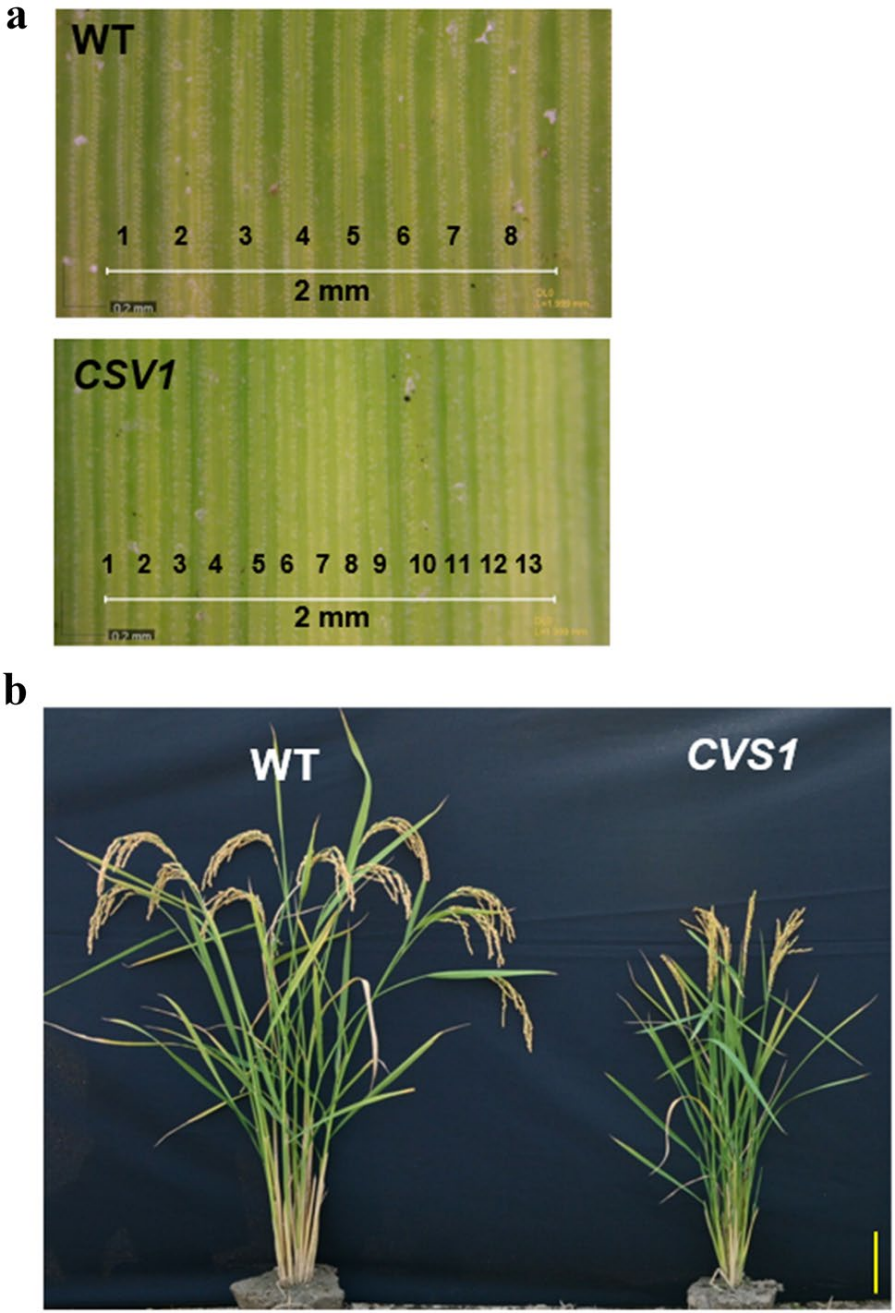
VD is increased but plant height is reduced in *CVS1*. **a** Representative images of leaf VD. Images were captured from fully expanded leaves of WT and *CVS1* plants of the *T*_2_ generation. The number on leaves indicates the vein number per 2 mm leaf width, with 8 veins in WT and 13 veins in *CVS1*. **b** Phenotypes of representative WT and *CVS1* plants of the *T*_4_ generation. The plant age was 120 days after sowing. Scale bar = 10 cm

In the *T*_1_ generation of *CVS1*, 2 out of 12 progenies exhibited the CVS phenotype, with an average VD of 7.00 ± 0.01 (Supplementary Fig. S2), which is consistent with the photo showing that the VD was 8 in WT and 13.5 in *CVS1* within a 2 mm leaf width (Fig. 2a). As VD was considered likely to exhibit phenotypic plasticity in response to environmental changes, the progeny of this mutant were advanced to successive generations through single seed decent by selecting only progenies with the highest VD in each generation until a predominant CVS phenotype was obtained (Supplementary Fig. S3). We did not observe a clear Mendelian inheritance in the early generations, which was attributed to the small population size screened and the lack of colinking information on T-DNA insertion and copy number in *CVS1*. However, by the *T*_6_ generation, progenies of *CVS1* could be clearly distinguished from the WT (Supplementary Fig. S3). *CVS1* also has a semidwarf stature and low grain yield phenotype compared to WT (Fig. 2b, Supplementary Fig. S6).

### Interveinal distance is reduced and M cell architecture is altered in *CVS1*

We found that compared with WT, vein number was increased by 2 ~ 3 per mm leaf width (~ 35%), and interveinal distance was reduced by 32% in *CVS1* (Fig. 2a, Table 1). There was no change in leaf thickness, BS cell number and area, or vein area. The average leaf width and bulliform cell area of *CVS1* were reduced by 37 and 40%, respectively (Table 1). Leaf VD was negatively correlated with leaf width in both WT and *CVS1* (Supplementary Fig. S4).

**Table 1:** Comparison of leaf anatomy between WT and *CVS1*

Parameter	Unit	WT	*CVS1*	*CVS1*/WT (%)
Vein density	Count per mm	**5.17 ± 0.17**	6.97 ± 0.24**	**135**
Leaf width	cm	0.83 ± 0.03	0.52 ± 0.06***	*63*
Leaf thickness	µm	95.19 ± 1.81	96.86 ± 3.20	102
Interveinal distance	µm	185.78 ± 2.75	126.16 ± 3.44***	*68*
M cell length	µm	24.61 ± 1.02	18.09 ± 0.51**	*74*
BS cell number	count	10.47 ± 0.54	10.24 ± 0.30	98
BS cell area	µm^2^	112.41 ± 11.42	97.84 ± 5.17	87
Vein area	µm^2^	779.19 ± 64.22	723.53 ± 62.77	93
Bulliform cell area	µm^2^	2117.42 ± 54.38	1278.16 ± 38.63***	*60*

Values are the means ± SE of measurements from 54 transverse leaf section images made on the left and right side of the leaf from three leaves of three *T*_6_ generation plants per line. *Represents a significant difference compared to WT, *P* ≤ 0.05, **P ≤ 0.01 and ***P ≤ 0.001. Bold and italics fonts indicate significant increases and decreases in value, respectively

**Fig. 3:**
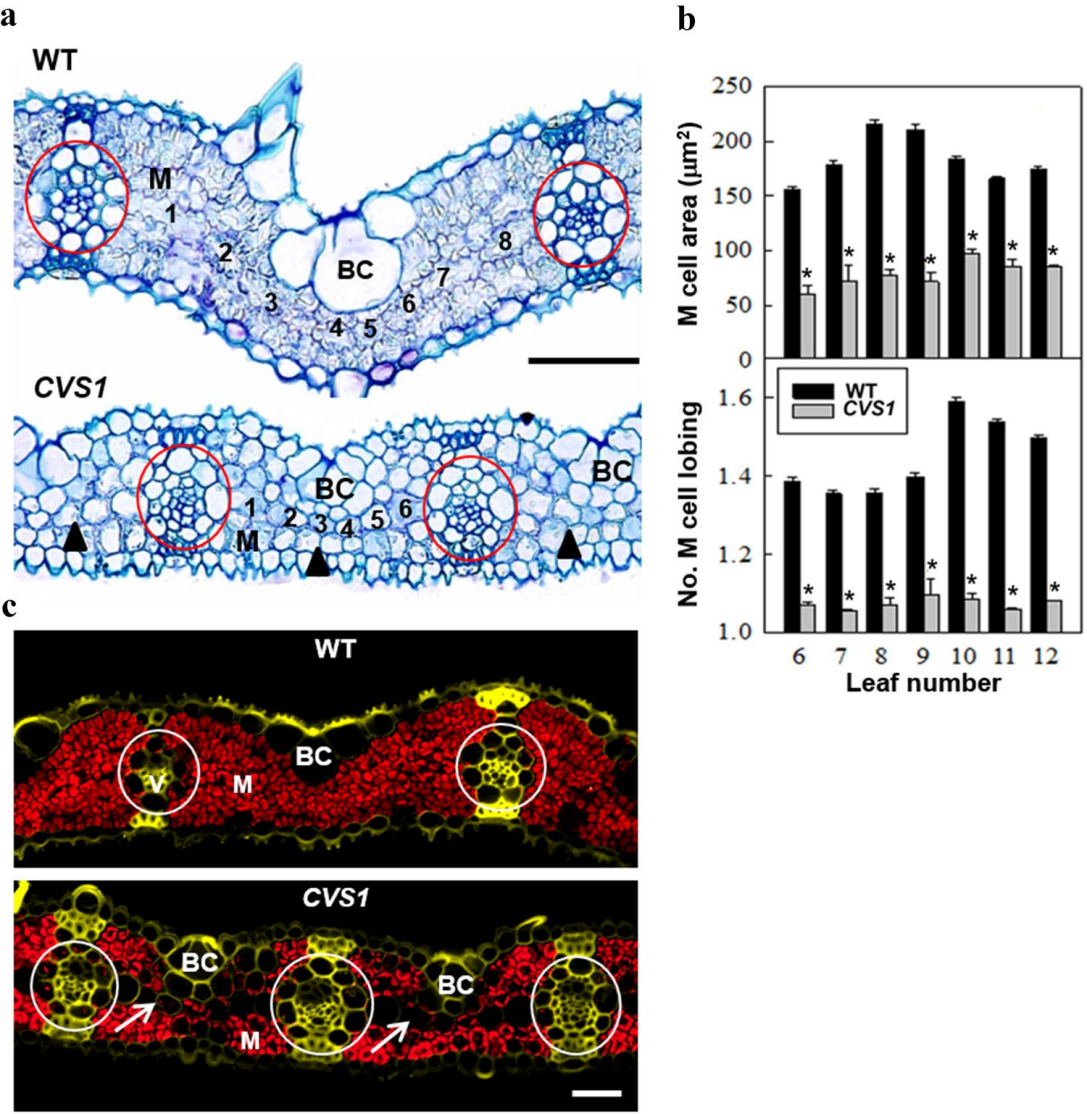
Interveinal spacing is reduced and M cell architecture is altered in *CVS1*. **a** Light micrographs illustrating transverse sections of M cells in the leaves of WT and *CVS1* plants of the *T*_2_ generation. M cell size was reduced in *CVS1*. Arrow heads indicate reduced M cell lobing in *CVS1*. Interveinal M cells are numbered. Scale bar = 50 µm. **b** Quantification of M cell area and lobing in leaves of WT and *CVS1*. Values are the average ± SE of measurements from a minimum of 25 random segments from 3 sections per leaf. Three leaves from three WT and *CVS1* plants of the *T*_4_ generation were measured. Asterisks denote significant differences compared to the WT (*P* < 0.005). **c** Chloroplast abundance in M cells in *CVS1*. Representative fluorescent images show transverse leaf sections of WT and *CVS1* plants of the *T*_6_ generation, depicting the red autofluorescence of chloroplasts. Arrows indicate reduced chloroplast content in M cells of *CVS1*. M *=* cells. B = Bulliform cells. Circles indicate vascular bundles. Magnification 200×. Scale bar = 20 µm

The CVS phenotype in *CVS1* was found to be associated with abnormal M cell development. There was a reduction in the length (by 26%) and total area of M cells (by 30–50%) (Table 1, Fig. 3a, b) in *CVS1*. A marked decline in M cell lobing in *CVS1* was also detected (Fig. 3a), with an average M cell lobing of 1.1 ± 0.01 in *CVS1* compared to 1.4 ± 0.04 in WT (Fig. 3b), which is accompanied by the abundance and positioning of chloroplasts (Fig. 3c). There was an almost complete absence of chloroplasts from M cells in the middle of leaves and an aggregation of chloroplasts around the periphery of M cells on both abaxial and adaxial leaf surfaces, suggesting that the development of chloroplasts was inhibited in *CVS1* (Fig. 3c).

### Photosynthetic performance is decreased in *CVS1*

**Fig. 4:**
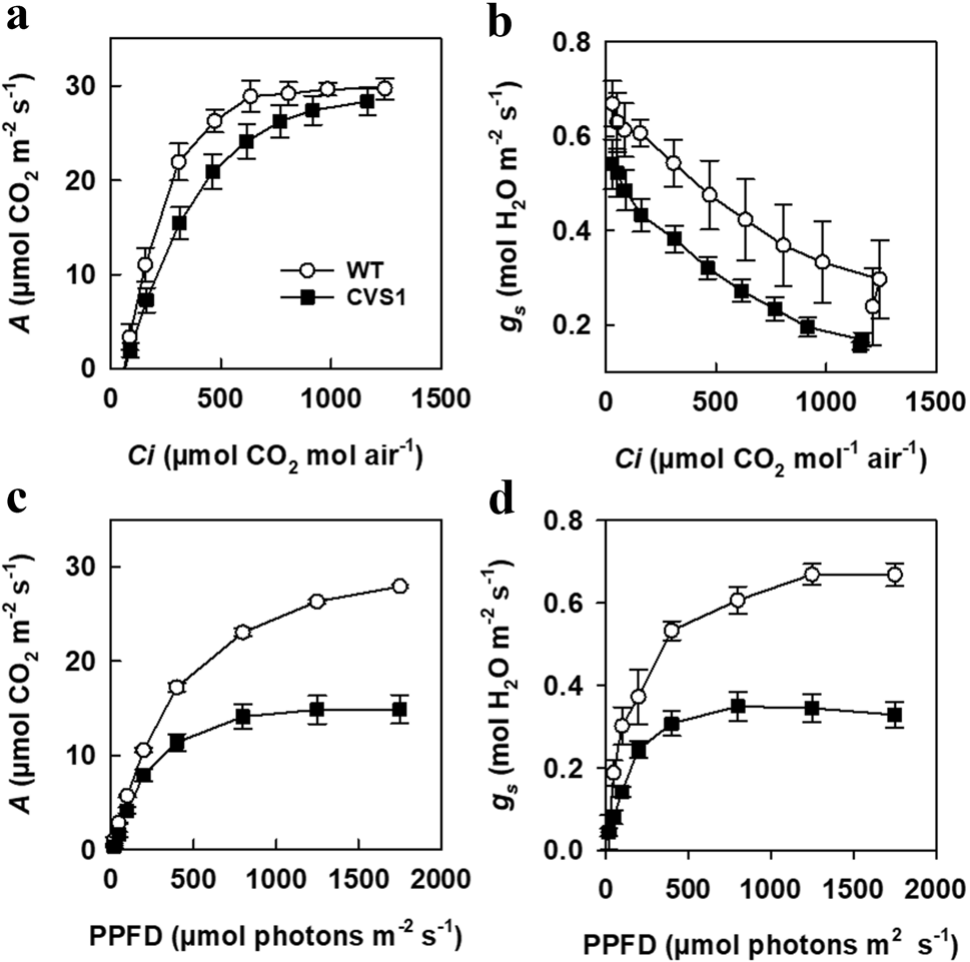
Photosynthetic performance is decreased in *CVS1*. **a** CO_2_ assimilation (A) and **b** conductance (gs) of WT and *CVS1* plants of the *T*_5_ generation and mutant **c** intercellular CO_2_ concentration (*Ci*; [left panel]) and **d** photosynthetic flux density (PPFD, [right panel]). Measurements were made at a leaf temperature of 30 °C and either a light intensity of 2,000 µmol photons m^−2^ s^−1^ or a *Ca* of 400 µmol mol^−1^ CO_2_. Values represent the mean ± SE of three leaves per plant and three plants per line

The rates of CO_2_ assimilation (*A*) were decreased in *CVS1* at all intercellular CO_2_ concentrations (*Ci*) (Fig. 4a) and stomatal conductance (*g*_s_) (Fig. 4c, d), reflecting a statistically significant lower *CE*, higher *R*_d_ and lower *g*_m_ (Table 2). There was no apparent difference in *V*_cmax_, *J* or *TPU* and no consistent statistically significant difference in *Γ*. The response of *A* to PPFD was also altered (Fig. 4b), with saturation of *A* occurring much earlier than normal at 750 µmol m^−2^ s^−1^ in *CVS1* compared to > 2000 µmol m^−2^ s^−1^ in WT. The quantum efficiency of CO_2_ assimilation in *CVS1* was also lower than the quantum efficiency of CO_2_ assimilation in WT (Table 2).

**Table 2:** Photosynthetic parameters of the wild-type (WT) and mutant *CVS1*

	φ	*Γ*	*CE*	*V*_cmax_	*J*	*TPU*	*R*_*d*_	*g*_*m*_
	μmol CO_2_ μmol^−1^ quanta	μmol CO_2_	μmol CO_2_ m^−2^ s^−1^ μmol CO2 mol^−1^	mmol m^−2^ s^−1^	mmol m^−2^ s^−1^	mmol m^−2^ s^−1^	mmol m^−2^ s^−1^	mmol m^−2^ s^−1^ Pa^−1^
WT	0.053 ± 0.000	59.5 ± 1.90	0.120 ± 0.009	131.02 ± 2.91	127.69 ± 4.74	9.33 ± 0.18	2.29 ± 0.26	3.30 ± 0.18
*CVS1*	0.042 ± 0.003	65.0 ± 3.20	0.080 ± 0.008***	136.72 ± 9.91	124.32 ± 7.59	9.02 ± 0.47	4.05 ± 0.41***	0.91*** ± 0.13***

Values are the means ± SE of one leaf from 3 plants per line. ***represents a significant difference compared to WT (*P* ≤ 0.005). *φ* quantum yield for CO_2_ assimilation, *Γ* CO_2_ compensation point, *CE* maximum carboxylation efficiency, *V*_*cmax*_ maximum carboxylation rate allowed by Rubisco, *J* rate of photosynthetic electron transport (based on NADPH requirements), *TPU* triose phosphate use, *R*_*d*_ daytime respiration, *g*_*m*_ mesophyll conductance

### Identification of T-DNA insertion sites in *CVS1*

**Fig. 5:**
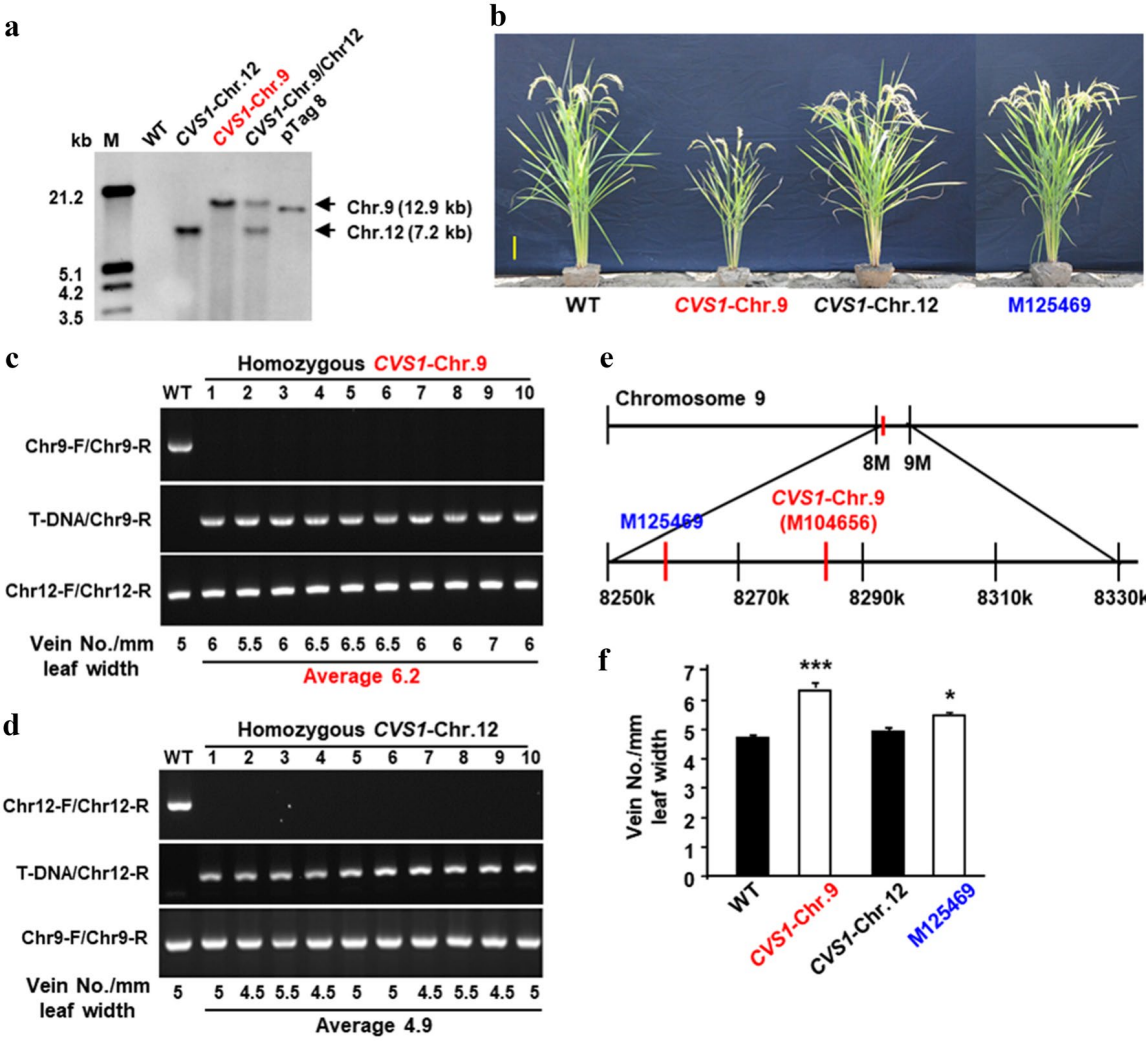
One of two T-DNAs inserted in the genome of *CVS1* is associated with the CVS phenotype. **a** DNA gel blot analysis using *Hyg* in the T-DNA as a probe. Plasimd *pTaq 8* was used as a positive control. M: molecular weight marker in kb. Only the T-DNA inserted on chromosome 9 was associated with the CVS phenotype. **b** Phenotypes of two segregated TRIM lines, *CVS1*-Chr.9 and *CVS1*-Chr.12, which each carry a T-DNA insertion on chromosomes 9 and 12, respectively. **c** Genotyping and phenotyping of homozygous *CVS1*-Chr.9. Three primer sets, Chr9-F and Chr9-R, T-DNA and Chr9-R, and Chr12-F and Chr12-R, amplified WT and T-DNA-inserted genomic DNA on chromosome 9 and WT genomic DNA of the T-DNA insertion site on chromosome 12. **d** Genotyping and phenotyping of homozygous *CVS1*-Chr.12. Three primer sets, Chr12-F and Chr12-R, T-DNA and Chr12-R, and Chr9-F and Chr9-R, amplified WT and T-DNA-inserted genomic DNA on chromosome 12 and WT genomic DNA of the T-DNA insertion site on chromosome 9. **e** T-DNA insertion sites of TRIM lines *CVS1*-Chr.9 (M0104656) and the allelic mutant M0125469 on chromosome 9. **f** Vein density of segregated *CVS1*-Chr.9 and *CVS1*-Chr.12 lines of the *T*_3_ generation and the allelic mutant M0125469. Values are the average ± SE of leaves from 10 plants of each line. Scale bar = 10 cm

Southern blot analysis of *CVS1* showed that two copies of T-DNA were inserted in the *CVS1* genome (Fig. 5a). The 2 T-DNA insertion sites were blasted to chromosomes 9 (locus 8,282,951 bp) and 12 (locus 22,42,9649 bp). The CVS and semidwarf phenotypes cosegregated with the T-DNA insertion on chromosome 9 but not the insertion on chromosome 12 (Fig. 5b–d) based on genotyping of homozygous lines (Fig. 5c, d). A more extensive genotyping analysis of *T*_5_ and *T*_6_ generations showed that both heterozygous and homozygous progenies possessed HVD morphology, which demonstrated that the CVS phenotype is likely due to a dominant mutation in *CVS1* (Fig. S5). Another TRIM line, M0125469, is a neighboring mutant of *CVS1* chromosome 9 (M0104656), as it contains a T-DNA inserted at a position 22 kb upstream of the T-DNA insertion site on chromosome 9 of *CVS1* (Fig. 5e). M0125469 exhibited a slightly higher VD than WT (Fig. 5f).

### Genes activated in *CVS1* and the allelic mutant M125469

**Fig. 6:**
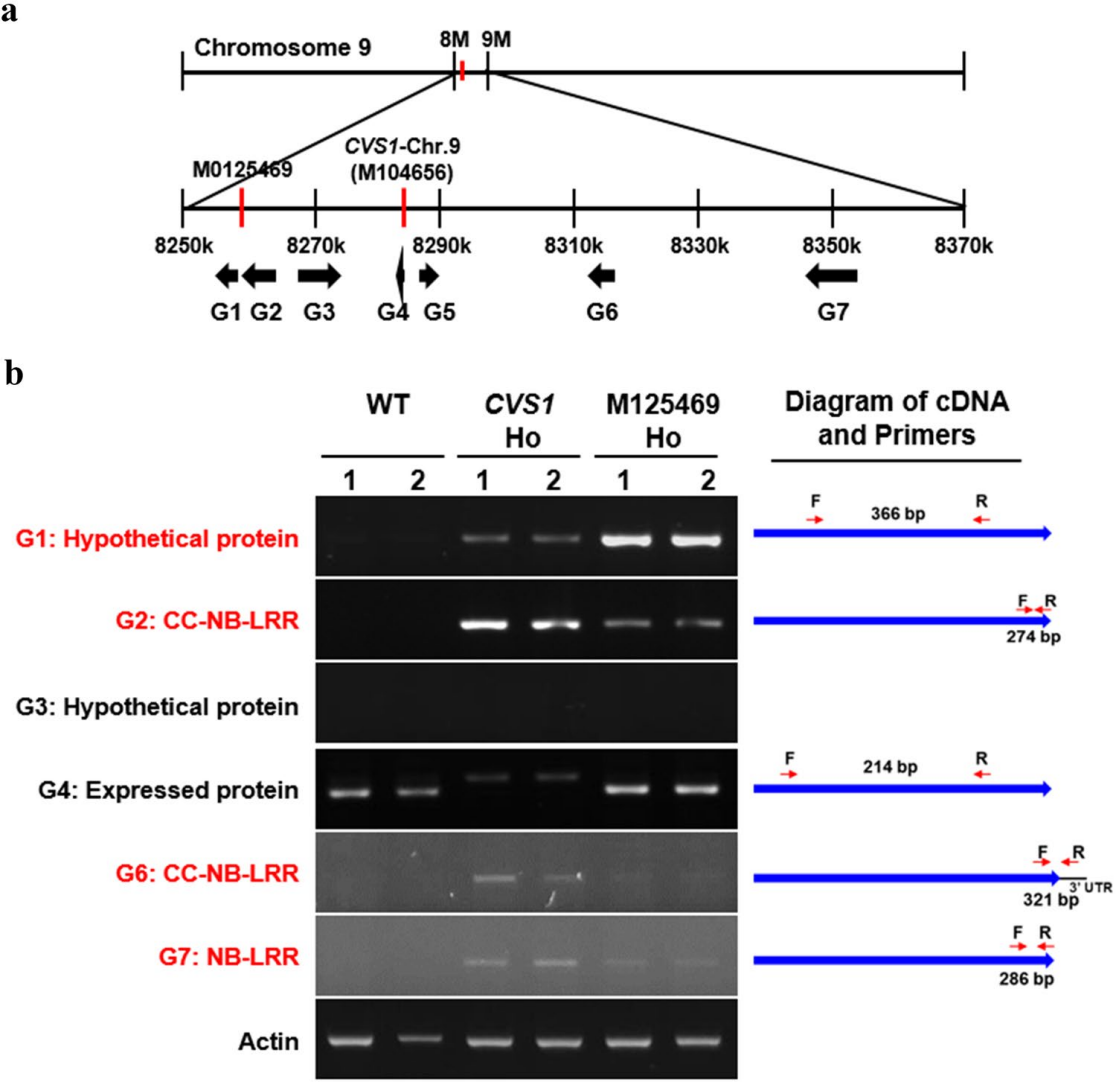
Expression of several genes flanking T-DNA is activated in *CVS1*. **a** Position of genes flanking T-DNA in *CVS1*. The T-DNA was inserted in chromosome 9 at 8,282,951 bp in *CVS1*. *G1*-*G7* indicate genes flanking T-DNA. **b** Transcript abundance of *G1*–*G7* in leaves of WT and *CSV1* plants of the *T*_6_ generation and the allelic mutant M0125469. The actin gene was used as an internal control. RNA samples were extracted from the 1st fully expanded leaf of two independent plants 80 days after imbibition. The right panel shows the diagram of cDNA and primer annealing regions for RT-PCR analysis. The red arrows indicate the direction and annealing regions of the primers

A total of 7 genes (designated G1–G7) were predicted to be present within a 70 kb region up- and downstream of the T-DNA insertion site on chromosome 9 of *CVS1* (Fig. 6a). These genes include hypothetical and NB-LRR proteins (Supplementary Table S4). Semiquantitative RT-PCR showed that only *G4* was expressed in WT leaves under normal growth conditions. *G5* is a putative transposon protein; thus, its expression was not analyzed. Expression of the *G1*, *G2*, *G6* and *G7* genes was activated in *CVS1*, expression of the *G4* gene was activated only in the allelic mutant M0125469 but not in *CVS1*, and expression of *G3* was not detected in any line (Fig. 6b). *G6* was not expressed in M0125469. T-DNA was inserted at a position 16 bp downstream of the stop codon within the 3’ untranslated region (3’UTR) of *G4*. We excluded *G4* from further analysis, as it was activated only in the allelic mutant M125469 and did not lead to a higher VD phenotype in *CVS1*.

### The CVS phenotype is recapitulated by overexpression of *NB-LRRs* in transgenic rice

**Fig. 7:**
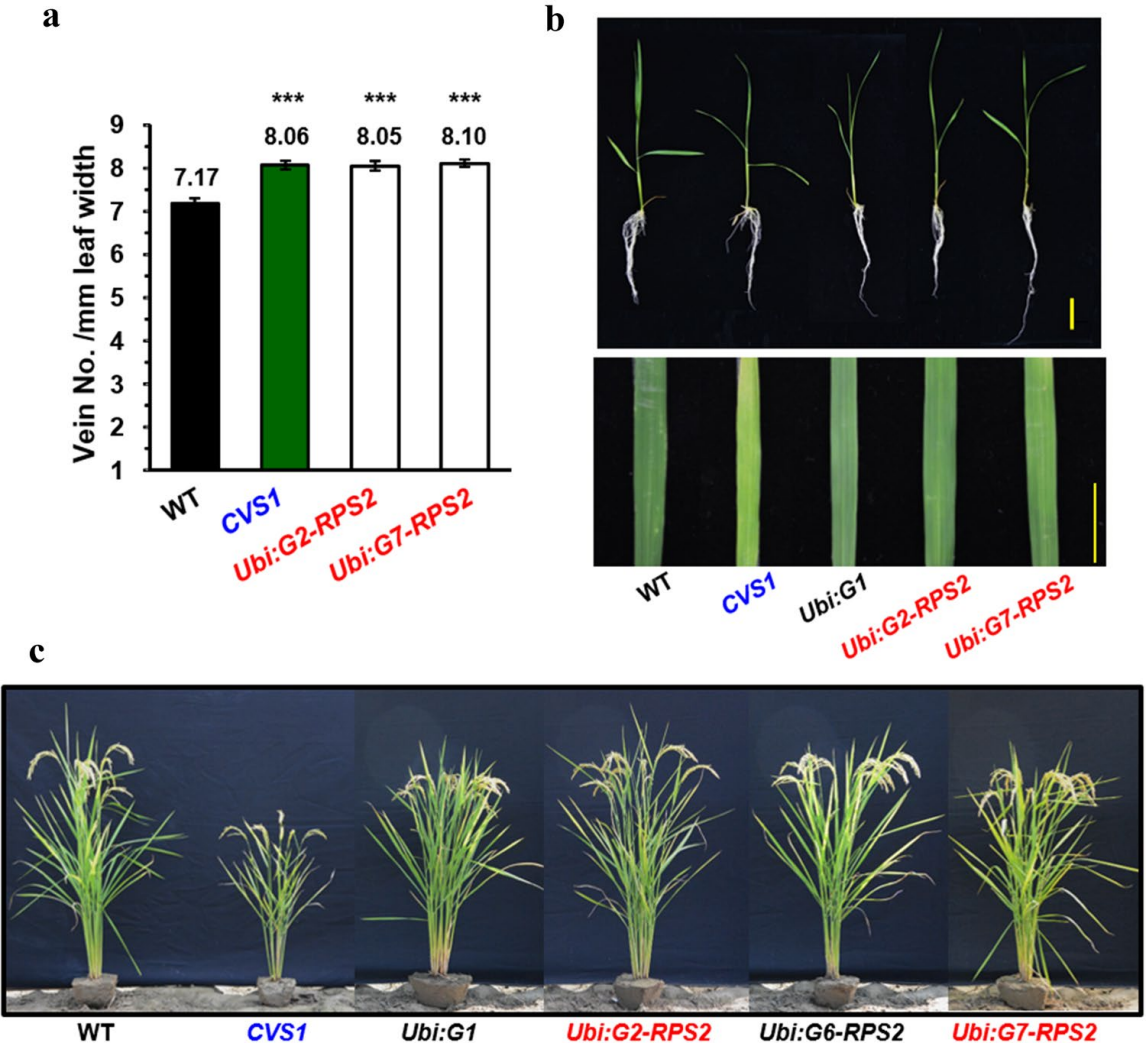
The CVS phenotype is recapitulated by overexpression of *NB-LRR* in transgenic rice. **a** Total vein numbers per leaf and vein numbers per mm leaf width. Values are the average ± SE of fully expanded leaves of 110-day-old plants. *n* = 18, 17, 39, 29, 40, 25 for WT, *CVS1*, and transgenic plants overexpressing the flanking genes *G1*, *G2*, *G6* and *G7*, respectively. **b** The seedling morphology, leaf color and width of 30-day-old transgenic rice seedlings overexpressing *Ubi:G2-NB-LRR* and *Ubi:G7-NB-LRR* were similar to the seedling morphology, leaf color and width of the WT. The scale of seedlings and leaves = 3 cm and 1 cm, respectively. **c** Mature transgenic rice plants overexpressing *Ubi:G2-NB-LRR* and *Ubi:G7-NB-LRR* grew similarly to the WT

**Fig. 8:**
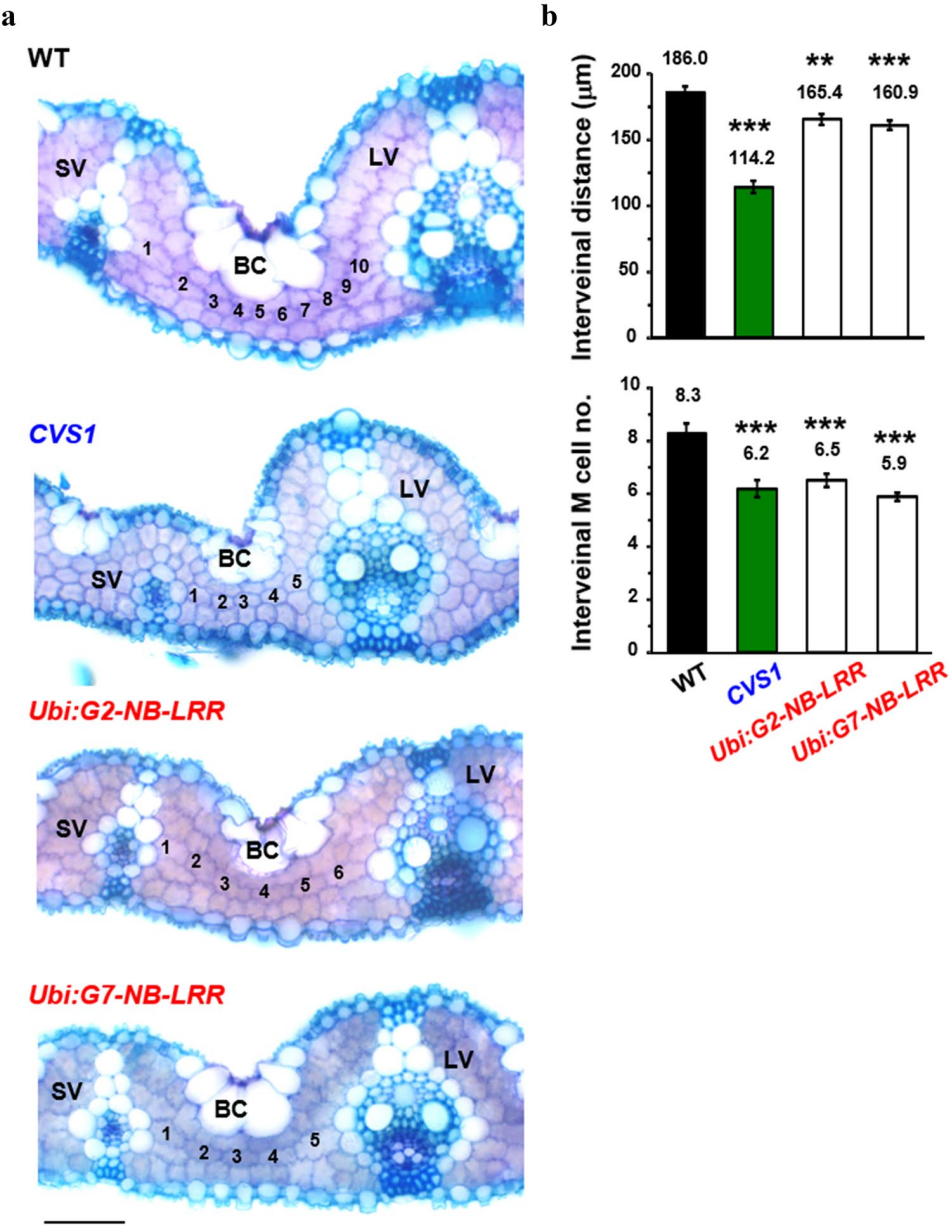
Transgenic plants overexpressing putative *RPS2* genes possess reduced interveinal spacing and M cell numbers. **a** Light micrographs illustrate transverse sections of leaf tissues of WT, *CVS1* (*T*_2_ generation) and transgenic rice carrying *Ubi:G2-NB-LRR* and *Ubi:G7-NB-LRR*. Numbers of M cells between two veins are labeled. SV: small vein, LV: large vein, BC: bulliform cells. Scale bar = 50 µm. b Interveinal spacing and M cell numbers were determined from the first fully expanded leaves of 20-day-old seedlings. *n* = 20, 19, 14, 29 for WT, *CVS1* and transgenic plants carrying *Ubi:G2-NB-LRR* and *Ubi:G7-NB-LRR*, respectively

*G1*, *G2*, *G6* and *G7* were individually overexpressed in transgenic rice under the control of the *Ubi* promoter. The mRNA of these genes accumulated into much higher levels in transgenic lines than in WT (Supplementary Fig. S7a–d). We found that only transgenic plants carrying the *Ubi:G2-NB-LRR* and *Ubi:G7-NB-LRR* constructs displayed the CVS phenotype; however, the increase in VD was not as high as that the increase in VD in *CVS1* (Fig. 7a, Supplementary Fig. S7e). We further screened the increase in VD at different stages and found that the increase in VD was highest at the earlier seedling stage, with 12% and 13% higher VD in *G2-NB-LRR and G7-NB-LRR* than in WT, respectively. As transgenic plants grow and mature, the increase in VD decreases from 12 and 13% to 4%. (Fig. 7a, Tables 3, 4). The *T*_3_ transgenic plants overexpressing *G2-NB-LRR* and *G7-NB-LRR* possessed slightly higher chlorophyll content and photosynthesis rate (the value of photosynthesis rate was not statistically significant), and limited to no yield penalty, thus the negative phenotypes of *CVS1* were eliminated (Supplementary Fig. S6). Seedlings and mature plants overexpressing *G2-NB-LRR* and *G7-NB-LRR* exhibited normal shoot and root growth, plant height, leaf width and leaf color in contrast to the semidwarf and narrow leaf phenotype in *CVS1* (Fig. 7b, c, Table 4). This study demonstrated that overexpression of two NB-LRRs increases VD without affecting plant growth from seedling to mature stages. As the seedlings of *G2-NB-LRR-* and *G7-NB-LRR*-overexpressing lines exhibited the highest increase in VD, we further characterized the pattern of M cells and bulliform cells for WT, *CVS1*, *G2-NB-LRR* and *G7-NB-LRR*-overexpressing lines. Reduced interveinal M cell numbers, M cell length and bulliform cell area were apparent in seedlings of *G2-NB-LRR-* and *G7-NB-LRR*-overexpressing lines, similar to those in *CVS1* (Fig. 8, Table 4). However, the M cell size and lobing were normal, which resulted in only a slight reduction in interveinal distances in these transgenic lines compared to WT (Fig. 8b).

**Table 3:** Comparison of vein density between WT, *G2-NB-LRR*, and *G7-NB-LRR* transgenic plants in different developmental stages

Stage	WT	*CVS1*	*G2-NB-LRR*	*G7-NB-LRR*	*G2*/WT (%)^a^	*G7*/WT (%)^a^
34 DAI seedling	7.17 ± 0.12	8.06 ± 0.10***^b^	8.05 ± 0.10***	8.10 ± 0.09***	112	113
50 DAI tillering	4.56 ± 0.11	6.29 ± 0.49**	4.90 ± 0.15	5.06 ± 0.14*	107	111
73 DAI reproductive	4.17 ± 0.06	5.55 ± 0.12***	4.56 ± 0.05**	4.52 ± 0.08**	109	108
114 DAI mature	4.24 ± 0.03	5.19 ± 0.08***	4.42 ± 0.04***	4.40 ± 0.04***	104	104

The transgenic plants of *G2-NB-LRR* and *G7-NB-LRR* are *T*_3_ generation. All plants were planted in the 2020-drying season, sample sizes (*n*) of WT, *CVS1*, *G2-NB-LRR* and *G7-NB-LRR* were 12, 12, 24, 24 for 34 DAI; 5, 4, 8, 9 for 50 DAI; 13, 14, 19, 21 for 73 DAI; and 24, 24, 48, 24 for 114 DAI, respectively

*DAI* days after imbibition

^a^*G2*/WT, *G7/WT* (%): WT was set as 100%, and the impact of *G2-NB-LRR* and *G7-NB-LRR* were calculated relative to this value

^b^Values are the means ± SE. Significance levels were determined with the *t* test: *P < 0.05, **P < 0.01, ***P < 0.001

**Table 4:** Comparison of leaf anatomy between WT and *G2-NB-LRR* and *G7-NB-LRR* transgenic plants at the seedling stage

Parameter of 34 DAI (seedlings)	Unit	WT	*CVS1*	*G2-NB-LRR*	*G7-NB-LRR*	*G2*/WT (%)	*G7/WT* (%)
Leaf width	mm	3.16 ± 0.05	2.77 ± 0.06**	3.01 ± 0.88	3.03 ± 0.06	95	96
Interveinal distance	µm	164.50 ± 4.82	118.00 ± 6.53**	124.62 ± 3.9***	130.20 ± 3.72***	76	79
Interveinal M cell no	count	6.32 ± 0.17	6.13 ± 0.12	5.16 ± 0.15***	5.56 ± 0.21*	82	88
M cell length	µm	30.83 ± 1.06	20.30 ± 0.78***	25.90 ± 0.5***	23.56 ± 0.54***	84	76
Bulliform cell area	µm^2^	3744.4 ± 183.7	2481.7 ± 88.2***	3009.9 ± 143.2***	2772 ± 152.1**	80	74

*G2*/WT, *G7/WT* (%): WT was set as 100%, and the impact of G2-NB-LRR and G7-NB-LRR were calculated relative to this value

Values are the means ± SE. Significance levels were determined with the *t* test: *P < 0.05, **P < 0.01, ***P < 0.001

The sample sizes (*n*) of WT, *CVS1*, *G2-NB-LRR* and *G7-NB-LRR* were 12, 12, 24, and 24 for vein density; 17, 13, 34, and 34 for interveinal distance; 43, 38, 32, and 34 for M cell no.; 53, 69, 108, and 105 for M cell length; and 9, 13, 15, and 15 for bulliform cell size, respectively

*DAI* days after imbibition

### NB-LRRs regulating M cell development evolutionarily diverge from other NB-LRRs regulating plant disease resistance

**Fig. 9:**
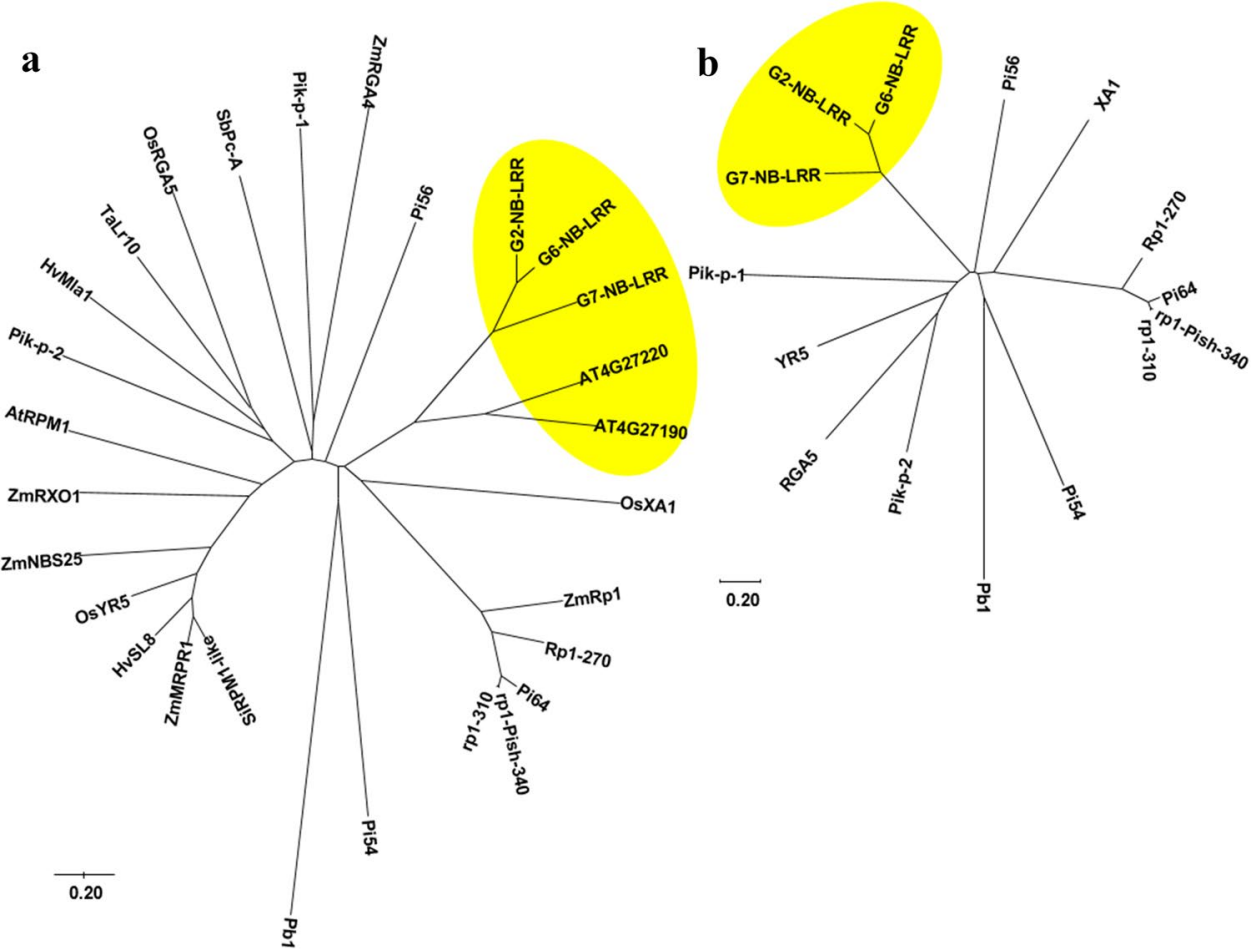
Phylogenetic analysis of NB-LRRs proteins in plants. **a** Phylogenetic analysis of 28 NB-LRRs from different plant species. The optimal tree with the sum of branch length = 17.06540678 is shown. **b** Phylogenetic analysis of 15 NB-LRRs from rice. The optimal tree with the sum of branch length = 10.33422275 is shown. Trees are drawn to scale, with branch lengths in the same units as the units of the evolutionary distances used to infer the phylogenetic tree. All ambiguous positions were removed for each sequence pair (pairwise deletion option). There were a total of 2044 positions in the final dataset. The scale value of 0.2 indicates 0.2 amino acid substitutions per site. The yellow background highlights the clade harboring G2-, G6- and G7-NB-LRR

Phylogenetic analysis of other rice NB-LRR proteins that have been reported to control disease resistance in rice shows that the three G2-, G6- and G7-NB-LRRs are classified into one distinct clade (Fig. 9). G2 and G6 are CC-NB-LRR-type proteins, and G7 is an NB-LRR-type protein (Supplementary Table S5). Amino acids of G2- and G6-NB-LRRs share higher identity and homology of 84 and 91%, respectively, with each other, compared to the identity and homology of 57–58 and 73–74%, respectively, with G7-NB-LRR (Supplementary Table S6). Surprisingly, G2-, G6- and G7-NB-LRRs share a very low identity and homology of less than 15 and 37%, respectively, with other NB-LRRs (Supplementary Table S6). Amino acid sequence alignment revealed the presence of conserved structural domains, i.e., NB and LRR, in G2-, G6- and G7-NB-LRRs and two other randomly selected rice NB-LRRs known to control disease resistance in rice (Supplementary Fig. S8). We further predicted the expression potential of *G2-, G6-, G7-NB-LRR*s and six other similar rice *NB-LRR* genes by GENEVESTIGATOR (v. 8.3.2) (Hruz et al. 2008), the expression potential of *G2-*, *G6-*, *G7-NB-LRRs* is relatively lower than the expression of the other six rice NB-LRRs in all developmental stages and different tissues, suggesting that these three *NB-LRRs* may play unique roles different from the other *NB-LRRs*.

## Discussion

### Genetic plasticity of VD is revalidated in rice

The highly efficient carbon fixation in leaves of C_4_ grasses relies partly on the combined anatomy of close vein spacing and functionally distinct photosynthetic cell types (Kumar and Kellogg 2019). Consequently, the increased VD is one of the key factors laying the foundation of a C_4_ anatomy in rice (Feldman, et al. 2014; Kajala et al. 2011; Langdale 2011). In rice, an increase in VD can be achieved by increasing vein number with no alteration in leaf width or by reducing M cell number to bring the BS cells surrounding two adjacent veins closer. In the present study, the VD of the TRIM mutant population ranged between 2.5 and 10 veins per mm leaf width. This result is significantly different from the result of WT TNG67, which consistently has a VD range between 4 and 6 veins per mm leaf width (Fig. 1). Heritable changes in VD in the TRIM population revalidated the genetic control of this trait in rice, as has been proposed (Feldman et al. 2014). However, a high number (53%) of apparently false-positive candidates indicated environmental control over vein development (Sack and Scoffoni 2013). The CVS phenotype identified in ~ 47% of mutant lines was associated with seed sterility or lethality, which made it difficult to screen for more mutants for the identification of genes regulating VD and related anatomical traits (Supplementary Table S3). Fortunately, *CVS1* showed a stably inherited increase in 2–3 veins per mm leaf width over WT, which clearly indicated that VD can be increased in rice (Fig. 2a, Table 1). *CVS1* provides a foundation to study aberrations in M cell structure in rice. Despite having negatively impacted traits such as leaf width, photosynthesis and growth, which appear similar to those in other rice VD mutants identified earlier (Smillie et al. 2012), *CVS1* plants were viable and produced seeds.

CVS in rice is a primary requirement for introducing Kranz anatomy in the leaf. To date, a few genes regulating leaf width and leaf rolling have been reported (Guo et al. 2019; Li et al. 2010; Qi et al. 2008; Schuler et al. 2018; Wang et al. 2016a, b), but no genes able to regulate VD without affecting yield have been identified in rice (Schuler et al. 2018; Sims et al. 2021). A leaf VD screen of the TRIM population was originally undertaken to understand genetic plasticity and identify genes regulating vein spacing development in C_3_ and C_4_ plants (Feldman et al. 2014; Luo et al. 2018). In the present study, we identified the *CVS1* mutant exhibiting a CVS phenotype with a gain-of-function C_4_–like leaf anatomy. Anatomical examination of M cells revealed that both reduced cell expansion and cell division account for the CVS phenotype in *CVS1*.

### High VD is associated with reduced interveinal M cell size and number in *CVS1*

A key feature of C_4_ leaf anatomy is an increased ratio of veinal to interveinal regions (Dengler et al. 1994; Muhaidat et al. 2007). *CVS1* showed a 35% increase in VD, because the interveinal space was reduced by ~ 32%, and an average reduction in individual M cell length by ~ 26% (Table 1). In a typical C_4_ leaf, veins are separated by 2–3 M cells compared to up to ~ 9 M cells in C_3_ leaves (Sheehy et al. 2008). Therefore, an ‘ideal’ rice mutant with Kranz-like internal leaf architecture would possess a significantly reduced internal M cell number. In the beginning of this study, we hypothesized that activation of gene expression could lead to changes in M cell number, a basis for the identification of VD mutants through the simple screening of a large mutant population. We found that *CVS1* shows a CVS phenotype due to a reduction in both M cell size and number (Figs. 3 and 8). Suggesting that both cell division and lateral M cell expansion have been affected in the mutant, in contrast to a study in which screening of a gene-deleted IR64 rice mutant population showed altered VD resulting from changes in M cell size rather than M cell number (Smillie et al. 2012), indicating that M cell development is under complex genetic control.

### Reduced M cell lobing and chloroplasts impair photosynthesis in *CVS1*

M cell lobing is a special characteristic of the chlorenchyma structure of rice and related warm-climate C_3_ grasses and has been implicated in refixation of the carbon that is lost during photorespiration in leaf tissues (Sage and Sage 2009). The presence of these specialized anatomical features is associated with high mesophyll conductance (Flexas et al. 2008; von Caemmerer et al. 2012a, b) and photosynthetic activity (Giuliani et al. 2013). The periphery of M cells with deep lobing is usually covered by chloroplasts and stromules to increase the cellular surface exposed to the intercellular airspace and maximize diffusive CO_2_ conductance and light transmission into the chloroplast stroma in rice (Giuliani et al. 2013; Sage and Sage 2009). Reduction of lobes, chloroplast number within M cells, and M cell size (Fig. 3) indicates a significant defectiveness in the coordinated development of chlorenchyma structure in *CVS1*. As a consequence, a reduction in the degree of M cell lobing was associated with reduced *g*_*m*_ and *A*, which resulted from a reduction in CO_2_ concentration within the intercellular air space and chloroplasts in *CVS1* (Fig. 4 and Table 2).

### NB-LRR proteins regulate M cell development in rice leaves

In the *CVS1* mutant, the multimerized *CaMV 35S* enhancers on the T-DNA led to the enhanced expression of three genes encoding *NB-LRR* and other genes of unknown functions. The three *NB-LRR* genes (*G2*, *G6* and *G7*) are present in a cluster on chromosome 9 and are activated to different extents by the T-DNA inserted in *CVS1* (Fig. 6). Transgenic rice overexpressing an Arabidopsis *NB-LRR* gene*, RPS2*, exhibits a semidwarf habit, fewer tillers per plant, and lower seed setting rate phenotypes (Li et al. 2019), which are similar to these seed setting rate phenotypes found in *CVS1* (Fig. 7c). Elevated accumulations of H_2_O_2_ and callose deposition are considered fitness costs for maintaining broad-spectrum resistance against pathogens and pests in transgenic rice overexpressing Arabidopsis *RPS2* (Li et al. 2019). However, in the present study, transgenic rice overexpressing the individual rice *NB-LRR* gene grew normally and limited to no yield penalty (Fig. 7b, c, Supplementary Fig. S6). It is unknown whether these rice *NB-LRR* genes have similar functions to Arabidopsis *RPS2* for conferring disease resistance. Interestingly, we found that only the overexpression of *G2-NB-LRR* or *G7-NB-LRR* led to an increased VD phenotype (Fig. 7a, Tables 3, 4), indicating that the regulation of vein development and plant growth is genetically separable. Auxin transporters play a crucial role in controlling vein development by auxin transport pathways (Sawchuk et al. 2013). The importance of auxin levels and polar transport in VD has been thoroughly discussed (Huang et al. 2017; Jiajia et al. 2020; Kumar and Kellogg 2019; Wang et al. 2017). Recently, the VD of *G2-NB-LRR* or *G7-NB-LRR* transgenic plants was found to increase significantly in the seedling stage, and the increase in VD decreased when the plants entered the reproductive stage (Table 3), consistent with the level of endogenous IAA, which is synthesized mainly in immature and meristematic tissues (Kasahara 2016; Leyser 2006). In most reports, auxin was classified as a negative regulator of innate immunity (Singh et al. 2018; Yang et al. 2013). A recent report showed that NB-LRR proteins activate multiple transcription factors via the regulation of auxin, JA and ET plant hormones to switch on defense responses under pattern-triggered immunity (PTI). NB-LRR proteins are the largest gene family and play pleiotropic roles in plants, such as cell growth, differentiation, signaling, and biotic and abiotic stress defense (Li et al. 2017; Meteignier et al. 2016; Uchida et al. 2011; Uchida and Tasaka 2011; Yang et al. 2010). The expression potentials of *G2- and G7-NB-LRRs* are also different from the expression potentials of other *NB-LRR* genes at all developmental stages (Fig. S9), indicating *G2- and G7-NB-LRRs* may have unique roles in rice. It is worthwhile to further study whether these *G2- and G7-NB-LRRs* have any functional link to the auxin signaling network.

Although overexpression of *G2-NB-LRR* or *G7-NB-LRR* increased VD in transgenic rice, the extent of the increase was much lower than the extent of the increase in *CVS1* (Supplementary Fig. S7e). One possibility is that overexpression of multiple genes that have been activated in the cluster on chromosome 9 is required for a significant increase in VD. This notion is supported by a study showing that constitutive overexpression of 60 known developmental regulators from maize individually did not confer an increased VD phenotype in transgenic rice (Wang et al. 2017). Alternatively, the leaf width and the interveinal distance were reduced by 37 and 32–34%, respectively, in *CVS1* compared with these leaf width and the interveinal distance in WT (Table 1, Fig. 8b), suggesting that a combination of the reduced leaf width and interveinal distance contributes to the significantly higher VD in *CVS1*. The chlorophyll content of transgenic rice overexpressing *G2-NB-LRR* and *G7-NB-LRR* is also slightly higher, but there is no significant difference in photosynthetic rate, which indicates that further introduction of C_4_ genes into transgenic rice with anatomical changes is necessary for functional C_4_ like rice creation (Ermakova et al. 2020; Sen et al. 2017). Studies with gene loss-of-function mutagenesis have identified numerous mutants with alterations in leaf anatomy associated with pleiotropic phenotypes (Fladung 1994; Rizal et al. 2015), suggesting that the establishment of C_4_ leaf anatomy is regulated by a complex regulatory network. Nevertheless, we found that the number but not the size of interveinal M cells was significantly reduced in the leaves of *G2-NB-LRR* and *G7-NB-LRR* transgenic rice plants, revealing an important factor controlling the interveinal distance in rice (Fig. 8, Table 4).

### Bulliform cell size also contributes to the CVS phenotype

The significant reduction in interveinal distance in *CVS1* also resulted from a combination of several internal morphological changes, including a reduction in M cell size and number and bulliform cell size (Fig. 8, Table 4). Bulliform cells are large, bubble-shaped epidermal cells that are present in groups on the adaxial surfaces of leaves in grasses. These cells are essential for water storage and are involved in the rolling of leaves to avoid water loss through transpiration under severe drought and salinity conditions (Grigore and Toma 2017). Loss of function of the *narrow leaf 7* (*NAL7*) gene, which controls auxin biosynthesis, results in reduced bulliform cell size and number and leaf width and slightly reduced interveinal distance, but not other phenotypes, in rice (Fujino et al. 2008). Supporting the notion that the phenotype of narrow leaves and reduced interveinal distance in *CVS1* could be related to bulliform cell size. *CVS1* and *G2-NB-LRR* and *G7-NB-LRR* transgenic rice exhibited increased VD and shortened interveinal distance by sharing altered phenotypes of reduced M cell and bulliform cell sizes. However, reduced lobing in smaller M cells was not detected in the *G2-NB-LRR* and *G7-NB-LRR* transgenic lines, indicating that the development of lobing is likely associated with M cell development. It is unclear whether the narrow leaf width along with the semidwarf plant architecture found in *CVS1* are regulated by multiple genes flanking the T-DNA or by somatic mutation linked to the T-DNA insertion.

## Supplementary Information

Below is the link to the electronic supplementary material.

Supplementary file1 (DOCX 3154 kb)
