# Supplementary material for: Closer vein spacing by ectopic expression of nucleotide-binding and leucine-rich repeat proteins in rice leaves
Source: Plant Cell Rep. 2021 Nov 27;41(2):319–35. doi: 10.1007/s00299-021-02810-5 (PMC8850240; doi:10.1007/s00299-021-02810-5)
Supplement: Supplementary file 1 — Supplementary file1 (DOCX 3154 kb) [file 299_2021_2810_MOESM1_ESM.docx]

**Closer vein spacing by ectopic expression of nucleotide-binding and leucine-rich repeat proteins in rice leaves**

Shun-Fang Lo ^1a^*^,^ Jolly Chatterjee ^2a^, Akshaya K Biswal ^2,3^, I-Lun Liu ^1^, Yu-Pei Chang ^1^, Pei-Jing Chen ^1^, Samart Wanchana ^2^, Abigail Elmido-Mabilangan ^2^, Robert Nepomuceno ^2^, Anindya Bandyopadhay ^2^, Yue-Ie Hsing ^4^, and William Paul Quick ^2,5^*

^1^ Biotechnology Center, National Chung Hsing University, Taichung 402, Taiwan, ROC

^2^ C_4_ Rice Centre, International Rice Research Institute (IRRI), Los Baños, Philippines.

^3^ Genetic Resources Program, International Maize and Wheat Improvement Center (CIMMYT), Carretera México-Veracruz km. 45, El Batán, Texcoco, México, C.P. 56237.

^4^ Institute of Plant and Microbial Biology, Academia Sinica, Taipei 115, Taiwan, ROC

^5^ Department of Animal and Plant Sciences, University of Sheffield, UK.

^a^ Equal contributors.

*cocorresponding authors

Shuen-Fang Lo

Phone: 886-4-2785-7739

Fax: 886-4-2285-7739

e-Mail: jjpipilo@gmail.com

ORCiD: 0000-0003-2278-6262

William Paul Quick

Phone: +63 2 8580 5600

Fax: +63 288450606

e-Mail: [w.p.quick@irri.org](mailto:w.p.quick@irri.org)

Authors’ email addresses

Shun-Fang Lo jjpipilo@gmail.com

Jolly Chatterjee j.chatterjee@irri.org

Akshaya K Biswal a.k.biswal@cgiar.org

I-Lun Liu druse107@yahoo.com.tw

Yu-Pei Chang mail200077@yahoo.com.tw

Pei-Jing Chen yahui0212@yahoo.com.tw

Samart Wanchana samartw2006@gmail.com

Abigail Elmido-Mabilangan a.elmido-mabilangan@irri.org

Robert Nepomuceno ran016@gmail.com

Anindya Bandyopadhay anindya.biotech@yahoo.com

Yue-Ie Hsing bohsing@gate.sinica.edu.tw

William Paul Quick w.p.quick@irri.org

**Supplementary Information:**

**Supplementary Table S1.** Field experiments for screening leaf CVS mutants.

**Supplementary Table S2.** Primers used for genotyping, PCR and RT-PCR analyses, and plasmid constructions.

**Supplementary Table S3.** List of *CVS* mutants and their heritability in successive generations (T_1_ – T_4_).

**Supplementary Table S4:** Location and annotation of genes flanking the T-DNA insertion site in *CVS1.*

**Supplementary Table S5.** The rice NB-LRRs proteins.

**Supplementary Table S6.** The identity percentage among rice NB-LRRs proteins.

**Supplementary Figure S1.** Map of the T-DNA vector *pTag 8* in *CVS1*.

**Supplementary Figure S2.** VD of *CVS1* progenies.

**Supplementary Figure S3.** Pedigree depict segregation of the CVS phenotype in *CVS1*.

**Supplementary Figure S4.** A negative correlation between leaf width and VD.

**Supplementary Figure S5.**  The *CVS1* mutant phenotype is dominant.

**Supplementary Fig. S6.** Transgenic plants overexpressing *G2-NB-LRR* and *G7-NB-LRR* possess slightly higher chlorophyll content and similar photosynthesis rates and grain yields compared with WT plants.

**Supplementary Fig. S7.** Transcripts of genes flanking the T-DNA insertion site on chromosome 9 of *CVS1* are highly accumulated in transgenic rice.

**Supplementary Figure S8.**  Comparison of amino acid sequences among selected NB-LRR proteins.

**Supplementary Figure S9.** Predictions of the expression potential of *G2*-, *G6*-, *G7-NB-LRRs* and six other most similar rice *NB-LRRs* by GRNEVESTIGATOR 8.3.2.

**Supplementary Table S1.** Field experiments for screening leaf CVS mutants.

| Experiment | Year | Season | Location | Total number of lines screened | Number of replicate plants grown per line | Number of candidates |
| --- | --- | --- | --- | --- | --- | --- |
| 1 | 2009 | WS | Taiwan | 4,970 | 12 | 6 |
| 2 | 2010 | DS | Taiwan | 3,026 | 12 | 15 |
| 3 | 2010 | WS | Taiwan | 2,831 | 12 | 0 |
| 4 | 2011 | DS | Philippines | 96 | 20 | 0 |
| 5 | 2011 | DS | Taiwan | 2,412 | 12 | 65 |
| 6 | 2011 | DS | Philippines | 427 | 20 | 9 |
| 7 | 2011 | WS | Taiwan | 1,500 | 13 | 2 |
| 8 | 2012 | DS | Taiwan | 1,001 | 12 | 1 |
| 9 | 2012 | WS | Taiwan | 1,061 | 12 | 2 |



The total number of mutant lines screened, average number of replicate plants grown per line, and number of *CVS* candidates are shown for each experiment (WS: wet season, July to November; DS: dry season, January to June).

**Supplementary Table S2.** Primers used for genotyping, PCR and RT-PCR analyses, and plasmid constructions.

| **Description** | **Name** | **Sequence (5’- 3’)** |
| --- | --- | --- |
| **Primers for genotyping or flanking sequence identification** | | |
| Hygromycin | HPT-F | GGCACAGCACATCAAAGAGA |
|  | HPT-R | CCCTTACGCTGAAGAGATGC |
| ß-Glucuronidase | GUS-F | CTATTTCTTTGCCCTCGGAC |
|  | GUS-R | CCTGACCTATTGCATCTCCC |
| FST Chr. 9 | Chr.9-F | TGAGAGGAGGGATGGGAG |
|  | Chr.9-R | CCTGTTTGCATTGAGTATGACC |
| FST Chr. 12 | Chr.12-F | CGACGAGAGAAGAAAACATCAG |
|  | Chr.12-R | GCAAAAAGAGAAACGCACAAG |
| T-DNA | RB-Seq4 | GCAGCGTACGAAGAAGGCCA |
| **RT-PCR analysis of gene expression** | | |
| Expressed protein | F19 G1-RT-F | GAAGTGGAAGGCGCAGCA |
|  | F19 G1-RT-R | CCTCTCCCACTCGCCCAAT |
| LOC_Os09g14010 | F19 G2-RT-F | CCCTTCATCGTGTCCAATAGT |
|  | F19 G2-RT-R | CGAGGGCATAGTGGAAGAAA |
| Hypothetical protein | F19 G3-RT-F | TTCGAGGACCTGTCAGTGCC |
|  | F19 G3-RT-R | ATGCACCTCGCTGTTGATG |
| LOC_Os09g14019 | F19 G4-RT-F | TTTCGGGTGTGCTGCTATAC |
|  | F19 G4-RT-R | TGAAGCACAGACGGTTATGACT |
| Hypothetical protein | F19 G5-RT-F | TTCGCCTCTCGTCGTCGGA |
|  | F19 G5-RT-R | CACGAACGGAGGACACCAA |
| LOC_Os09g14060 | F19 G6-RT-F | CAGAGTCCATGCCTTCCCTAA |
|  | F19 G6-RT-R | GCGATAAAGGGATTGGTGCC |
| LOC_Os09g14100 | F19 G7-RT-F | CGTTCCAGTTCCAGCCAGAC |
|  | F19 G7-RT-R | GATGCCACCTGTGCTCCAAA |
| Actin 1 | Actin-F | CTTCATAGGAATGGAAGCTGCGGGTA |
|  | Actin-R | TTCCTGTGCACAATGGATGG |
| 18S rRNA | 18S-F | CCTCGTGCCCCTATCAACTT |
|  | 18S-R | GACACTAAAGCGCCCGGTAT |

RB, right boarder. FST, Flanking Sequence Tag.

**Supplementary Table S3.** List of *CVS* mutants and their heritability in successive generations (T_1_ – T_4_).

| Lines | Mutant | Year | Wet / Dry Season | Ratio of CVS:WT Phenotypes | | | | Comment |
| --- | --- | --- | --- | --- | --- | --- | --- | --- |
|  | Accession |  |  | T_1_ | T_2_ | T_3_ | T_4_ |  |
| 1 | M0020314 | 2009 | WS | 1:11 | - | - | - | Sterile |
| 2 | M0104656 | 2009 | WS | 2:12 | 16:49 | 25:94 | - | *CVS1** |
| 3 | M0105588 | 2009 | WS | 1:12 | 1:48 | 6:15 | - | *CVS2* |
| 4 | M0106332 | 2009 | WS | 1:12 | 0:22 | - | - |  |
| 5 | M0106602 | 2009 | WS | 1:12 | 0:23 | - | - |  |
| 6 | M0108615 | 2009 | WS | 2:12 | 0:22 | - | - |  |
| 7 | M0110124 | 2009 | WS | 1:12 | 2:18 | 7:28 | - |  |
| 8 | M0111167 | 2010 | DS | 1:11 | 0:22 | 0:30 | - |  |
| 9 | M0111211 | 2010 | DS | 1:12 | 0:19 | 0:30 | - |  |
| 10 | M0111350 | 2010 | DS | 9:11 | 0:20 | 0:30 | - |  |
| 11 | M0111374 | 2010 | DS | 1:12 | 0:20 | - | - |  |
| 12 | M0111536 | 2010 | DS | 1:12 | 2:19 | 0:31 | - |  |
| 13 | M0111572 | 2010 | DS | 1:12 | 0:18 | - | - |  |
| 14 | M0111610 | 2010 | DS | 2:11 | 0:20 | - | - |  |
| 15 | M0111680 | 2010 | DS | 1:10 | 0:03 | - | - |  |
| 16 | M0111816 | 2010 | DS | 2:09 | 0:20 | - | - |  |
| 17 | M0112652 | 2010 | WS | 2:10 | 2:15 | - | - |  |
| 18 | M0112738 | 2010 | DS | 1:12 | 0:20 | - | - |  |
| 19 | M0112856 | 2010 | DS | 1:10 | 2:19 | 0:30 | - |  |
| 20 | M0113198 | 2010 | DS | 1:11 | 0:20 | - | - |  |
| 21 | M0113623 | 2010 | DS | 1:12 | 0:18 | - | - |  |
| 22 | M0114187 | 2010 | DS | 1:08 | 0:18 | - | - |  |
| 23 | M0114331 | 2010 | DS | 1:12 | 1:17 | 0:30 | 0:30 |  |
| 24 | M0114437 | 2010 | DS | 1:11 | 0:20 | - | - |  |
| 25 | M011455 | 2010 | DS | 1:12 | 0:20 | - | - |  |
| 26 | M0114760 | 2010 | WS | 2:12 | - | - | - | Died |
| 27 | M0115016 | 2010 | DS | 1:11 | 0:05 | - | - |  |
| 28 | M0115463 | 2010 | DS | 1:11 | 0:05 | - | - |  |
| 29 | M0115641 | 2010 | WS | 2:12 | - | - | - | Died |
| 30 | M0116043 | 2010 | WS | 1:12 | - | - | - | Died |
| 31 | M0116411 | 2010 | WS | 4:12 | - | - | - | Died |
| 32 | M0116427 | 2010 | WS | 1:12 | - | - | - | Sterile |
| 33 | M0117076 | 2010 | WS | 2:12 | - | - | - | Died |
| 34 | M0118094 | 2010 | WS | 4:12 | - | - | - | Sterile |
| 35 | M0118227 | 2011 | WS | 1:12 | - | - | - | Sterile |
| 36 | M0118522 | 2011 | WS | 1:12 | - | - | - | Sterile |
| 37 | M0119633 | 2011 | WS | 1:12 | - | - | - | Sterile |
| 38 | M0121451 | 2011 | WS | 1:12 | - | - | - | Sterile |
| 39 | M0122794 | 2011 | WS | 1:12 | - | - | - | Sterile |
| 40 | M0123767 | 2011 | WS | 2:10 | - | - | - | Sterile |
| 41 | M0124008 | 2011 | WS | 2:11 | - | - | - | Sterile |
| 42 | M0124384 | 2011 | WS | 1:12 | - | - | - | Sterile |
| 43 | M0124669 | 2011 | WS | 1:12 | - | - | - | Sterile |
| 44 | M0124738 | 2012 | DS | 1:12 | - | - | - | Sterile |
| 45 | M0125000 | 2012 | DS | 2:12 | - | - | - | Sterile |
| 46 | M0125026 | 2012 | DS | 1:11 | - | - | - | Sterile |
| 47 | M0125439 | 2012 | DS | 1:11 | - | - | - | Sterile |
| 48 | M0125797 | 2012 | DS | 1:12 | - | - | - | Sterile |
| 49 | M0126437 | 2012 | DS | 1:12 | - | - | - | Sterile |

Lines labeled in red exhibited the CVS phenotype at the T_3_ generation.

*: Germination is poor in *CVS1* mutants, therefore the ratio phenotype (CVS:WT) cannot represent the ratio of genetic segregation.

**Supplementary Table S4:** Location and annotation of genes flanking the T-DNA insertion site in *CVS1.*

| **Gene code** | **Accession** | | **Putative function** | | **Distance from T-DNA in Kb** | **Database** | |  |  |
| --- | --- | --- | --- | --- | --- | --- | --- | --- | --- |
| G1 |  | | hypothetical protein | | 22 | RiceGAAS | |  |  |
| G2 | LOC_Os09g14010 | | CC-NB-LRR | | 14.9 | RGAP7 | |  |  |
| G3 |  | | hypothetical protein | | 12 |  | |  |  |
| G4 | LOC_Os09g14019 | | expressed protein | | 0.25 | RGAP7 | |  |  |
| G5 |  | | hypothetical protein | | 3 |  | |  |  |
| G6 | LOC_Os09g14060 | | CC-NB-LRR | | 33.1 | RGAP7 | |  |  |
| G7 | LOC_Os09g14100 | | NB-LRR | | 71.3 | RGAP7 | |  |  |
|  | |  | |  | | |  | |  |

**Supplementary Table S5:** The rice NB-LRR proteins*.*

| Gene | MSU locus | Protein type | Amino acid number |
| --- | --- | --- | --- |
| NB-LRRs from rice | |  |  |
| CVS-G2 | LOC_Os09g14010 | CC-NBS-LRR | 1016 |
| CVS-G6 | LOC_Os09g14060 | CC-NBS-LRR | 895 |
| CVS-G7 | LOC_Os09g14100 | NBS-LRR | 991 |
| Pi56 | LOC_Os09g16000 | NBS-LRR | 743 |
| XA1 | LOC_Os04g53120 | CC-NBS-LRR | 1885 |
| Rp1-270 | LOC_Os01g57270 | CC-NBS-LRR | 1273 |
| Pi64 | LOC_Os01g57280 | CC-NBS-LRR | 1327 |
| Rp1-340 | LOC_Os01g57340 | CC-NBS-LRR | 1290 |
| Rp1-310 | LOC_Os01g57310 | CC-NBS-LRR | 1290 |
| Pi54 | NA | NBS-LRR | 330 |
| Pb1 | LOC_Os11g37050 | CC-NBS-LRR | 1420 |
| Pik-p-2 | LOC_Os11g46210 | NBS-LRR | 1044 |
| RGA5 | LOC_Os11g11810 | CC-NBS-LRR | 1301 |
| YR5 | LOC_Os11g12330 | NBS-LRR | 905 |
| Pik-p-1 | LOC_Os11g46200 | CC-NBS-LRR | 1125 |
| NB-LRRs from different plant species | |  |  |

**Supplementary Table S6:** The identity percentage among rice NB-LRR proteins

|  | G2-NB-LRR | G6-NB-LRR | G7-NB-LRR | Pi56 | XA1 | Rp1-270 | Pi64 | Rp1-340 | Rp1-310 | Pi54 | Pb1 | Pik-p-2 | RGA5 | YR5 | Pik-p-1 |
| --- | --- | --- | --- | --- | --- | --- | --- | --- | --- | --- | --- | --- | --- | --- | --- |
| CVS-G2 | 100 | 91 | 74 | 31 | 30 | 42 | 25 | 32 | 27 | 27 | 27 | 27 | 32 | 30 | 31 |
| CVS-G6 |  | 100 | 73 | 33 | 32 | 42 | 27 | 32 | 30 | 30 | 30 | 29 | 32 | 31 | 30 |
| CVS-G7 |  |  | 100 | 32 | 32 | 44 | 26 | 33 | 28 | 28 | 27 | 27 | 31 | 30 | 31 |
| Pi56 |  |  |  | 100 | 39 | 39 | 26 | 23 | 28 | 28 | 28 | 28 | 30 | 28 | 29 |
| XA1 |  |  |  |  | 100 | 41 | 30 | 25 | 28 | 28 | 28 | 29 | 33 | 29 | 28 |
| Rp1-270 |  |  |  |  |  | 100 | 29 | 32 | 32 | 32 | 32 | 32 | 35 | 33 | 31 |
| Pi64 |  |  |  |  |  |  | 100 | 25 | 23 | 23 | 22 | 23 | 21 | 22 | 21 |
| Rp1-340 |  |  |  |  |  |  |  | 100 | 25 | 25 | 25 | 25 | 24 | 28 | 26 |
| Rp1-310 |  |  |  |  |  |  |  |  | 100 | 99 | 94 | 79 | 36 | 36 | 29 |
| Pi54 |  |  |  |  |  |  |  |  |  | 100 | 94 | 79 | 36 | 36 | 29 |
| Pb1 |  |  |  |  |  |  |  |  |  |  | 100 | 80 | 35 | 36 | 29 |
| Pik-p-2 |  |  |  |  |  |  |  |  |  |  |  | 100 | 36 | 34 | 27 |
| **OsRGA5** |  |  |  |  |  |  |  |  |  |  |  |  | **100** | **36** | **28** |
| **OsYR5** |  |  |  |  |  |  |  |  |  |  |  |  |  | **100** | **27** |
| **Pik-p-1** |  |  |  |  |  |  |  |  |  |  |  |  |  |  | **100** |

**
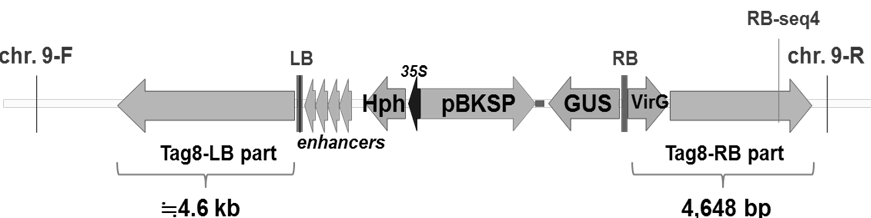
**

**Supplementary Figure S1. Map of the T-DNA vector *pTag 8* in *CVS1*.**

*pTag 8* T-DNA vector was used for activation tagging. LB and RB, left and right border of T-DNA, respectively; enhancers, 4 enhancers of the cauliflower mosaic virus 35S promoter (*CaMV35S*) gene; *Hph*, hygromycin phosphotransferase gene; *p35S*, promoter of cauliflower mosaic virus 35S promoter (*CaMV35S*) gene (-343 to -46 bp upstream of the enhancers); *pBKSP,* backbone of plasmid pBluescript; and *GUS*, *b*-glucuronidase cDNA. *VirG*, partial nucleotide sequence of the virulence gene *virG* of the *Agrobacterium* Ti plasmid; the distance between RB and LB of T-DNA is approximately 8.4 kb. Extra ~4.6 kb backbone residues were introduced into the genome of *CVS1* outside both LB and RB.

**
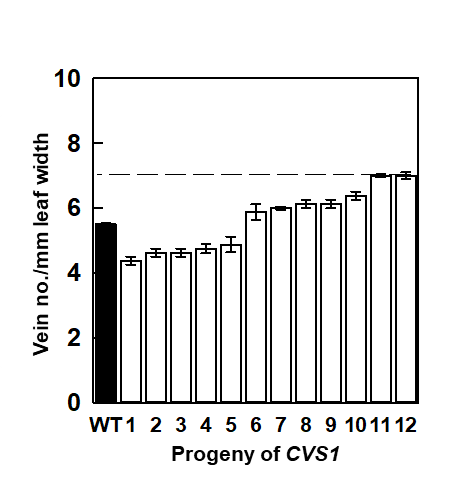
**

**Supplementary Figure S2. VD of *CVS1* progenies.**

VD of *CVS1* progenies of the T_1_ generation. Values are the average ± SE of 4 VD counts from a single fully expanded leaf of individual mutant progenies, and the average ± SE of 12 WT plants. Plants with a VD of 7 (dashed line) or more were classified as *CVS* mutants.

**
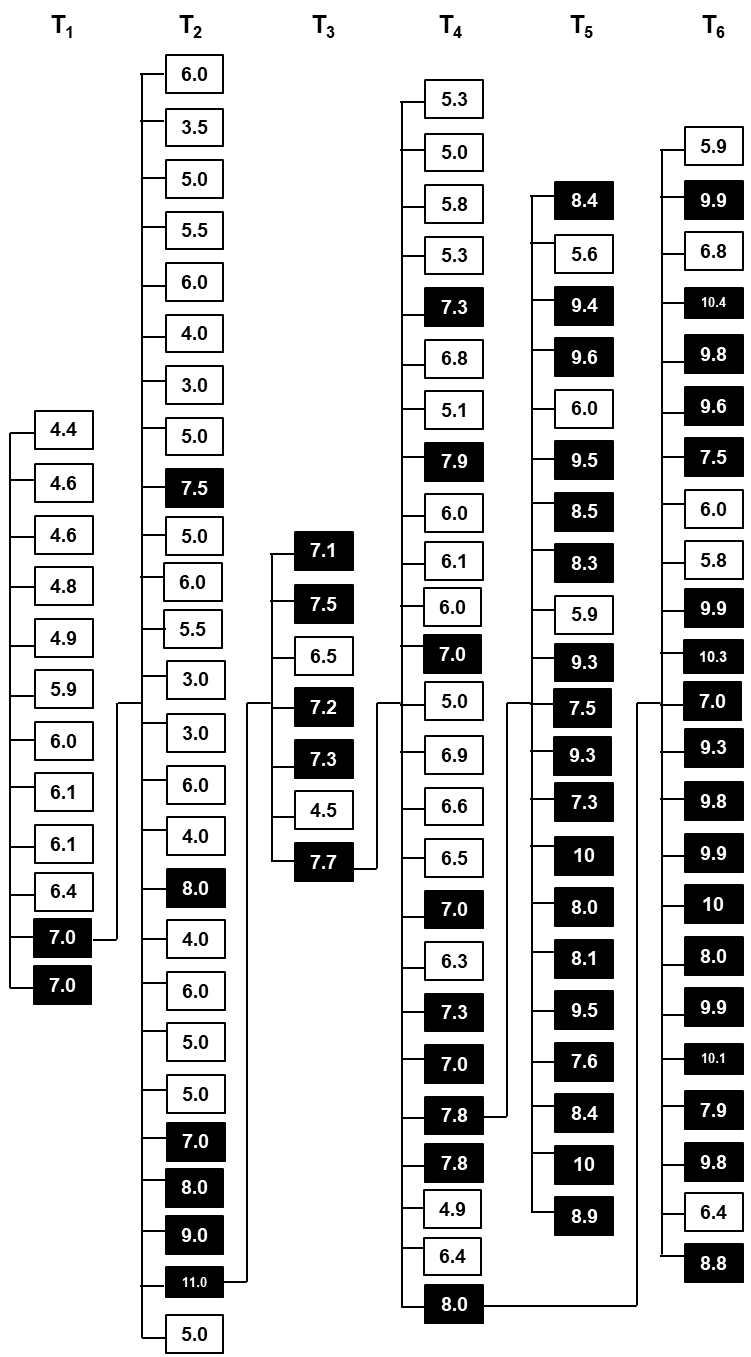
**

**Supplementary Figure S3. Pedigree depict segregation of the CVS phenotype in *CVS1*.**

The pedigree traces the segregation of the CVS phenotype from the T_1_ to the T_6_ generations of *CVS1* progenies. Black boxes denote progenies with the CVS phenotype. The number inside the boxes is the number of veins per mm leaf width.

**
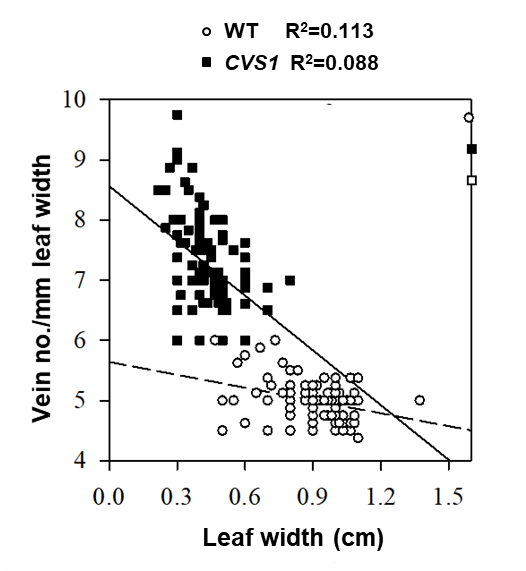
**

**Supplementary Figure S4. A negative correlation between leaf width and VD.**

Values are the average vein density (VD) in plants of 117 WT and 117 progenies of *CVS1* of the T_1_ to T_4_ generations.

**
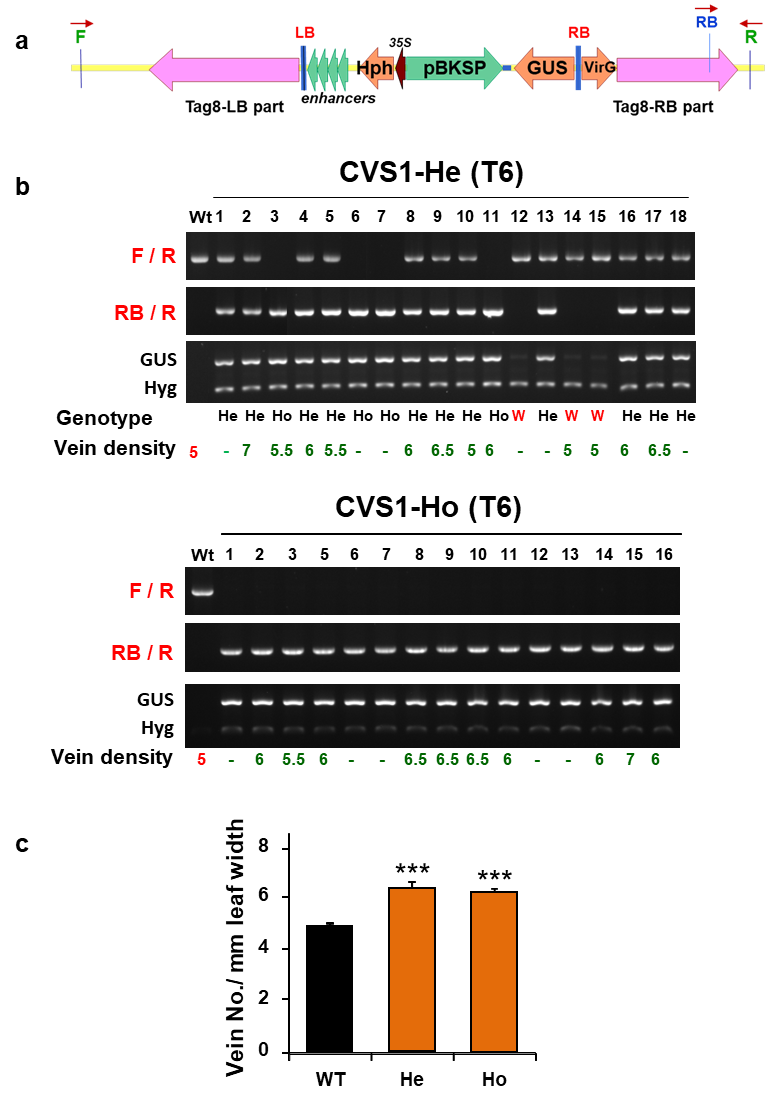
**

**Supplementary Figure S5. The *CVS1* phenotype is dominant.**

(a) Diagram of T-DNA vector inserted in the *CVS1* mutant, F, R, and RB are the primers for genotyping of T-DNA insertion site. The red arrows above F, R, and RB indicate the direction of primer annealing.

(b) Genotyping of *CVS1* T6 progenies.

(c) The vein density of *CVS1* T_5_ progenies showed both heterozygous and homozygous progenies possessing the HVD morphology. Total vein numbers per mm leaf width are shown. Values are the average ± SE of fully expanded leaves of 110-day-old plants. n = 100, 8, and 23 for WT, *CVS1-He* and *CVS1-Ho* plants. ***: P<0.001.

**
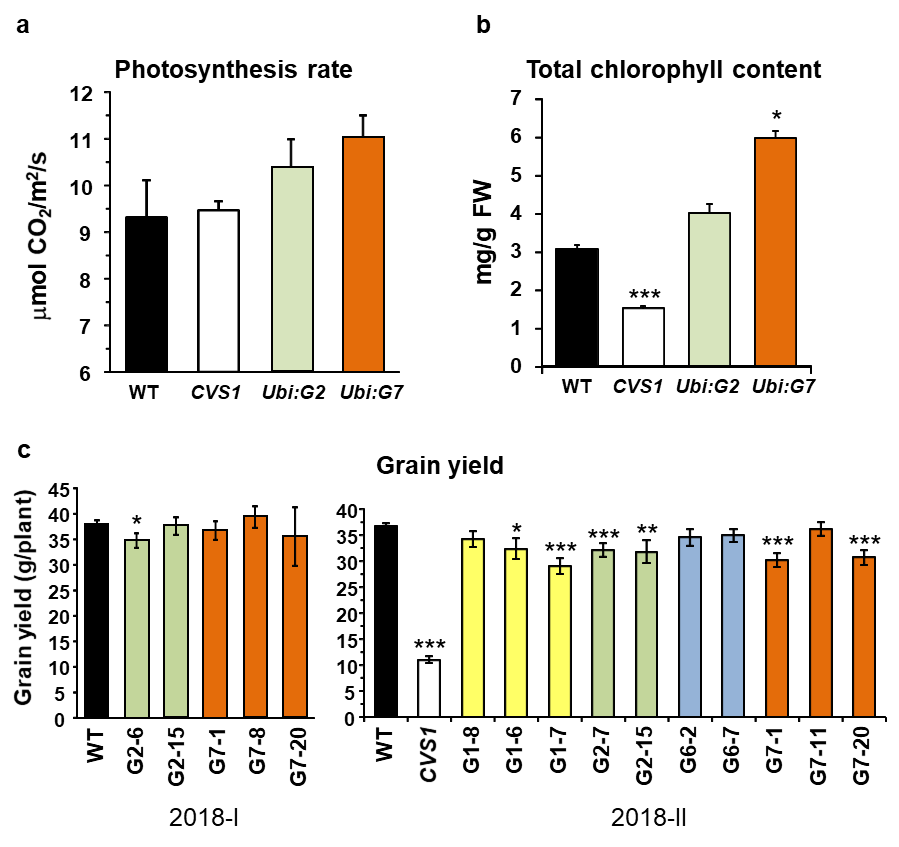
**

**Supplementary Figure S6. Transgenic plants overexpressing *G2-NB-LRR* and *G7-NB-LRR* possess** **slightly higher chlorophyll content, similar photosynthesis rate and limited to no yield penalty compared with those of WT.**

(a) Photosynthesis rate, (b) chlorophyll content, and (c) total grain yield per plant from the 2018-I and 2018-II cropping seasons.

The sample numbers for photosynthesis rate, and chlorophyll content were 5 and 6 for WT, *CVS1* and all individual lines of transgenic plants overexpressing flanking genes *G1*, *G2*, *G6* and *G7*, respectively. For the grain yield, the sample numbers of WT for 2018-I and 2018-II were 84 and 4, respectively, and 18 for *CVS1* and all transgenic lines overexpressing flanking genes *G1*, *G2*, *G6* and *G7*

**
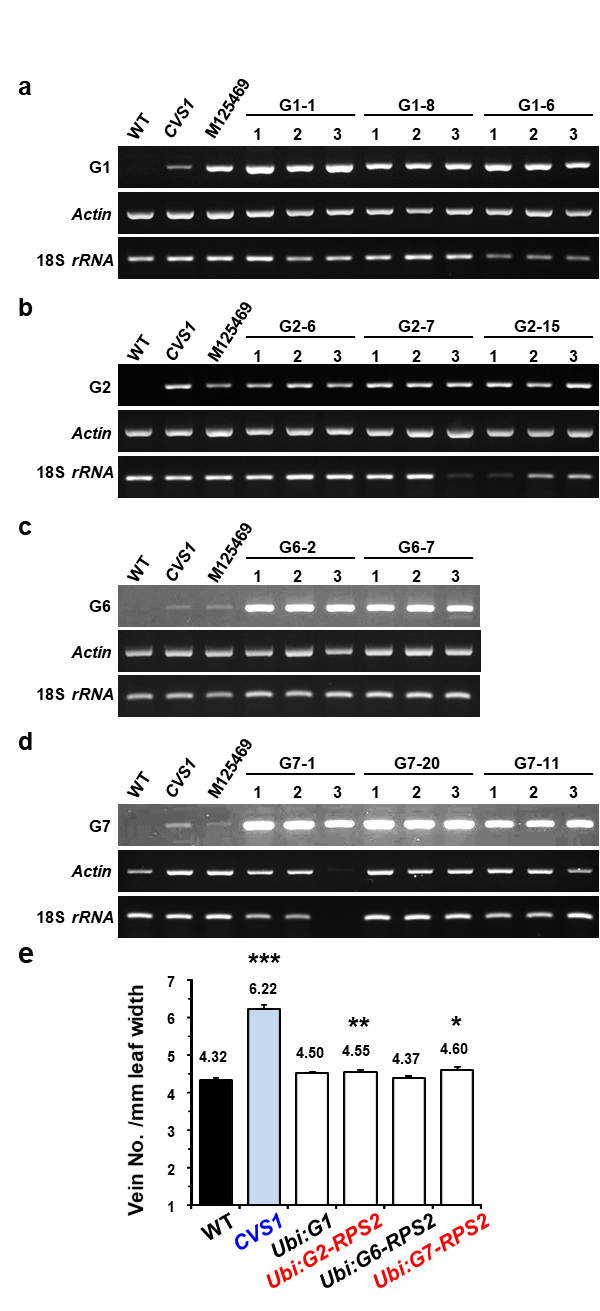
**

**Supplementary Figure S7. Transcripts of genes flanking the T-DNA insertion site on chromosome 9 of *CVS1* are highly accumulated in transgenic rice, and the CVS phenotype is recapitulated by overexpression of *G2* and *G7-NB-LRRs* in transgenic rice.**

RT-PCR analyses of RNAs extracted from leaves of 3-week-old seedlings of transgenic lines overexpressing (a) *G1*, (b) *G2*, (c) *G6* or (d) *G7*. Three individual plants of three independent transgenic lines were used for the extraction of total RNA. *CVS1* and allelic mutant M125460 were included as control lines. *Actin* and 18S r*RNA* were used as control genes. (d) Total vein numbers per leaf and vein numbers per mm leaf width. Values are the average ± SE of fully expanded leaves of 110-day-old plants. n = 18, 17, 39, 29, 40, 25 for WT, *CVS1*, and transgenic plants overexpressing the flanking genes *G1*, *G2*, *G6* and *G7*, respectively.

**
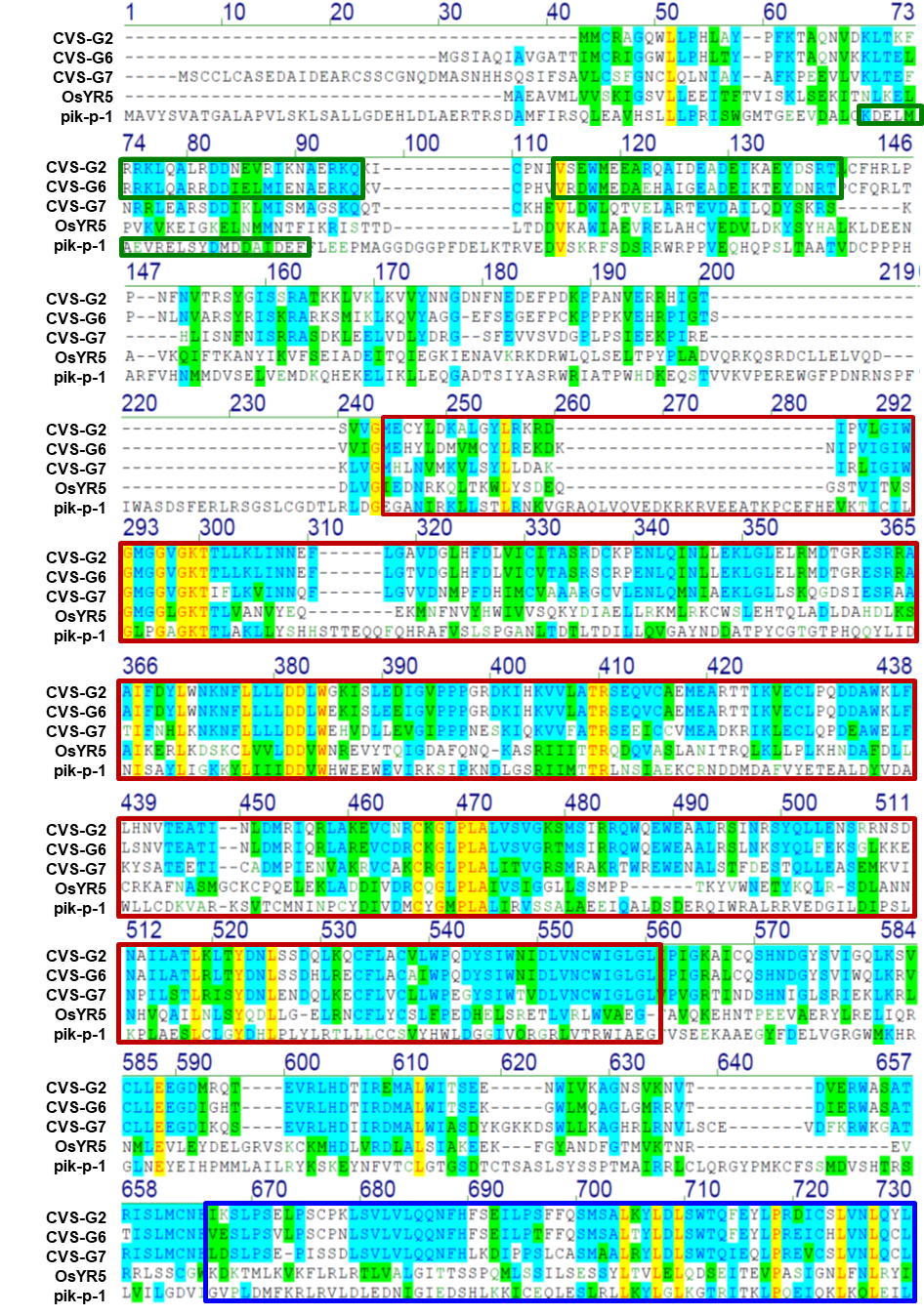
**

To be continued

**
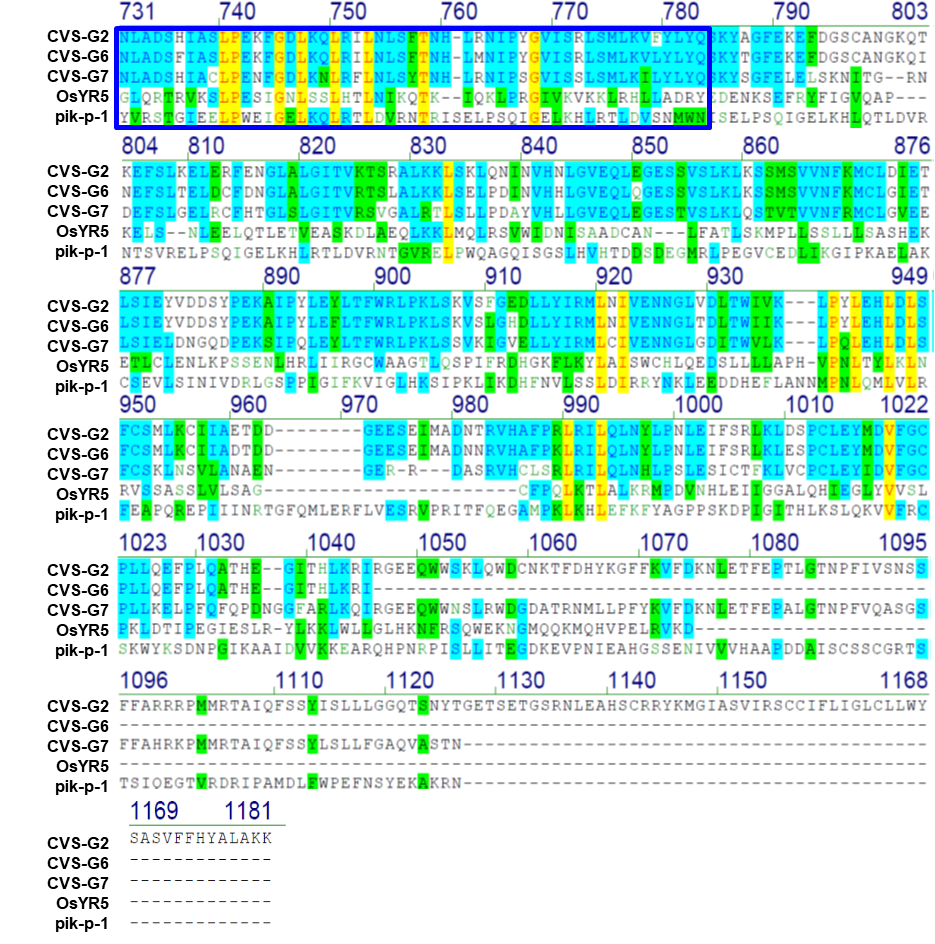
**

**Supplementary Figure S8. Comparison of amino acid sequences among selected NB-LRR proteins.**

Amino acid sequence alignment was conducted using Vector NTI 11.0 software, and various functional domains were predicted with MSU Rice Genome Annotation Project Release 7 software (Kawahara et al., 2013). The green box indicates the N-terminal coiled-coil (CC) domain, red box indicates the central nucleotide-binding (NB) domain, and the blue box indicates the C-terminal leucine-rich repeat (LRR) domain.

**
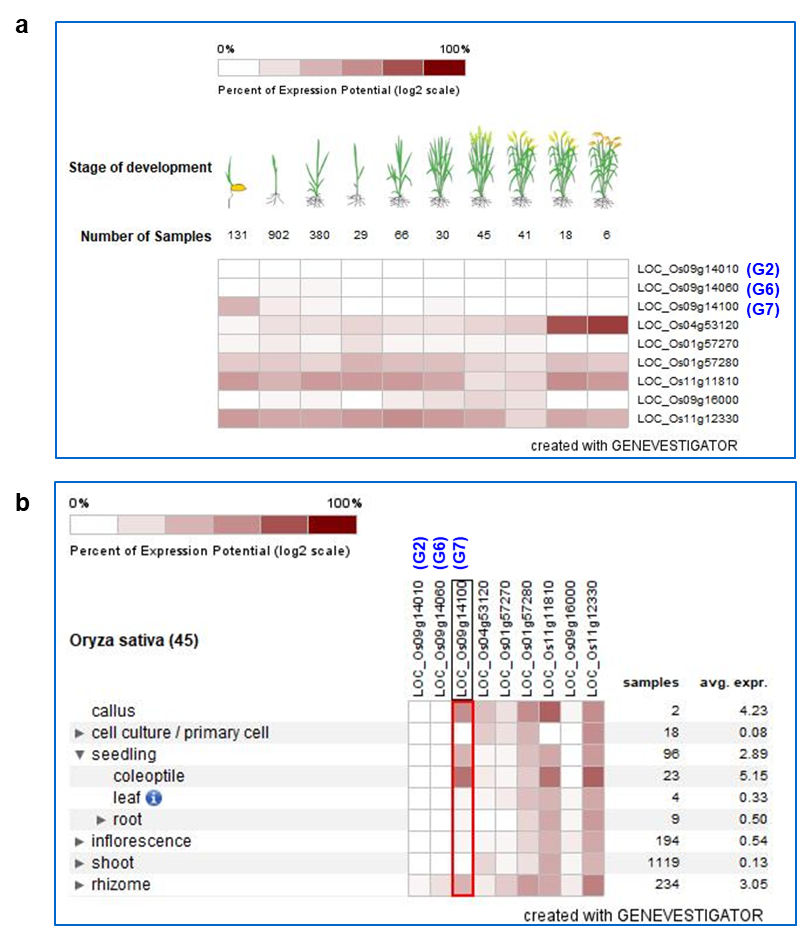
**

**Supplementary Figure S9. Predictions of the expression potential of *G2*-, *G6*-, *G7-NB-LRRs* and six other similar rice *NB-LRRs* by GRNEVESTIGATOR 8.3.2.**

1. Expression predictions based on developmental stages.
2. Expression predictions by specific tissues/organs**.**
